# Autoprocessing and oxyanion loop reorganization upon GC373 and nirmatrelvir binding of monomeric SARS-CoV-2 main protease catalytic domain

**DOI:** 10.1038/s42003-022-03910-y

**Published:** 2022-09-16

**Authors:** Nashaat T. Nashed, Daniel W. Kneller, Leighton Coates, Rodolfo Ghirlando, Annie Aniana, Andrey Kovalevsky, John M. Louis

**Affiliations:** 1 Laboratory of Chemical Physics, National Institute of Diabetes and Digestive and Kidney Diseases, National Institutes of Health, DHHS, Bethesda, MD 20892-0520 USA; 2 Neutron Scattering Division, Oak Ridge National Laboratory, 1 Bethel Valley Road, Oak Ridge, TN 37831 USA; 3 Second Target Station, Oak Ridge National Laboratory, 1 Bethel Valley Road, Oak Ridge, TN 37831 USA; 4 Laboratory of Molecular Biology, National Institute of Diabetes and Digestive and Kidney Diseases, National Institutes of Health, DHHS, Bethesda, MD 20892-0520 USA

**Keywords:** Enzyme mechanisms, X-ray crystallography, Proteases, Viral infection, Target validation

## Abstract

The monomeric catalytic domain (residues 1–199) of SARS-CoV-2 main protease (MPro^1-199^) fused to 25 amino acids of its flanking nsp4 region mediates its autoprocessing at the nsp4-MPro^1-199^ junction. We report the catalytic activity and the dissociation constants of MPro^1-199^ and its analogs with the covalent inhibitors GC373 and nirmatrelvir (NMV), and the estimated monomer-dimer equilibrium constants of these complexes. Mass spectrometry indicates the presence of the accumulated adduct of NMV bound to MPro^WT^ and MPro^1-199^ and not of GC373. A room temperature crystal structure reveals a native-like fold of the catalytic domain with an unwound oxyanion loop (E state). In contrast, the structure of a covalent complex of the catalytic domain-GC373 or NMV shows an oxyanion loop conformation (E* state) resembling the full-length mature dimer. These results suggest that the E-E* equilibrium modulates autoprocessing of the main protease when converting from a monomeric polyprotein precursor to the mature dimer.

## Abstract (web summary)

Structural characterization and catalytic activity of SARS-CoV-2 main protease reveal minimal interface regions enabling dimer formation driven by inhibitor-induced conformational changes of the oxyanion loop.

## Introduction

The spacio-temporal regulation of virally encoded proteases and ordered processing of viral polyproteins into functional units is indispensable for the assembly of the replication/transcription complex and production of viable progeny virion^1–5^. In Severe Acute Respiratory Syndrome CoronaVirus 2 (SARS-CoV-2) and its closely related SARS-CoV, a single copy of the main protease (MPro, nsp5) encoded within the polyprotein (pp) 1a and 1ab mediates its self-cleavage (autoprocessing) at its termini (nsp4/nsp5 and nsp5/nsp6) and other cleavages between nsp6 and nsp16^1–7^. Thus, the mature MPro has been at the forefront for drug development and several lead compounds, including nirmatrelvir (NMV) that has recently received emergency use authorization by the U. S. Food and Drug Administration for the treatment of COVID-19, targeting the active site have been described^3,8–12^. In this regard, a detailed understanding of MPro release from its precursor polyprotein provides an attractive target for structure and mechanism-based design of active site and dimerization inhibitors prior to its maturation.

The fully active mature MPro is a homodimer, with one active site per monomer, and exhibits a monomer-dimer dissociation constant (*K*_dimer_) in the low micromolar range^3,13^. The active site consists of a Cys145-His41 catalytic dyad^14^. Each monomer contains 306 amino acids that make up three domains, I-III. Domains I (residues 8–101) and II (residues 102–184) exhibit a chymotrypsin-like fold, and domain III (residues 201–306), comprises a cluster of five alpha-helices connected to domain II by a long loop (residues 185–200)^3,7^. Notably, domain III is present only in viruses belonging to the order Nidovirales^15^, which includes coronaviruses.

Similar to a picornavirus 3C-like cysteine protease^16,17^ which contains the two domains similar to domains I and II of MPro, MPro exhibits substrate specificity for the sequence (Leu/Ile)-Gln↓(Ser/Ala/Gly), where ↓ indicates the site of cleavage^7,18^. The mechanism and intermediates produced in the maturation process of MPro and their catalytic activities are not fully understood. In vitro studies of the precursor are complicated to carry out because a single copy of MPro is anchored on either side by membrane spanning regions within nsp4 and nsp6 flanking MPro (nsp5). It is conceivable, however, that an ensemble of folding intermediates between two MPro chains may mediate transient cleavages at its termini during the early steps of the polyprotein processing cascade. Cleavage at the N-terminus of MPro^7,19–21^ has been proposed to modulate the *K*_dimer_ and ensuing catalytic activity through conformational rearrangements by forming inter and intra monomer contacts of the free N-terminal residues with domains II and III. Consequently, deletion of the N-terminal residues 1–7 (termed the N-finger) or domain III leads to a major shift in the monomer-dimer equilibrium mainly to the monomer form accompanied by a drastic decrease in mature-like catalytic activity^17,22–24^. Various mutational analyses of SARS-CoV MPro and structural requirements for its regulation are summarized in references^25,26^. Despite the monomer form adopting a native-like tertiary fold, as shown for various mutations or deletions in the sequence, monomeric variants of MPro are reported to exhibit very low or no catalytic activity^25,26^. This has been attributed to an altered active site that occludes binding of Q-P1 of the substrate in the S1 subsite leading to loss of catalytic function^25,27–29^. Substrate induced dimerization as a pathway to its maturation and catalytic function has been proposed^30,31^. The interdependency of dimerization to catalytic activity based on numerous mutational studies is summarized in reference^26^. In this context, the S5 loop residues Q189-A194 and domain III undergo a significant conformational rearrangement upon N-terminal cleavage and dimerization^25,27^. All of the above studies pertain to the previous SARS-CoV isolate.

Mutations of critical dimer interface residues such as G11, S139, E290 and R298 have been shown to result in significantly increasing the *K*_dimer_^7,27–29,32^. In recent studies we demonstrated the modulation of the monomer-dimer equilibrium of a full-length MPro monomeric construct (MPro^M^), bearing 2 substitution mutations (E290A and R298A) in domain III, by a transition state analog inhibitor GC373 (the reactive aldehyde form of GC376)^13^. The results provide conclusive evidence that the appearance of mature-like catalytic activity was dependent on dimer formation with two equivalent active sites. In this model system, dimerization and inhibitor binding were inseparable events. It also pointed to a conformational active site equilibrium switching between an inactive state (E) and an active state (E*) synchronized with dimerization, particularly that of the N-terminal dimer interface region and domain III reorientation, the active E* state being dominant in the wild-type MPro (MPro^WT^).

In this study, we examined the physical properties, catalytic activities, and room temperature crystal structures of MPro analogs comprising the catalytic domain and the connecting loop up to residue 199. Therefore, these constructs could serve as early mimetics of a folded intermediate of MPro in the polyprotein form with an exclusion of the entire helical domain III. They are monomeric and display catalytic activities and N-terminal autoprocessing exclusive for a monomeric form as compared to that of the dimeric MPro^WT^. The X-ray structure of the monomeric catalytic domain is similar to the dimer but with an unwound oxyanion loop conformation. In contrast, the binding of GC373 and NMV restores the conformation of the oxyanion loop to that of the active dimeric enzyme. Minimal interface regions enabling dimer formation upon inhibitor binding were identified and related to the stability of the enzyme-inhibitor complex. Importantly, these studies also conclusively show that inhibitors designed to bind to mature MPro dimer also bind to its monomer form.

## Results and discussion

### Characterization and catalytic activity of monomeric MPro^1–199^

**Fig. 1:**
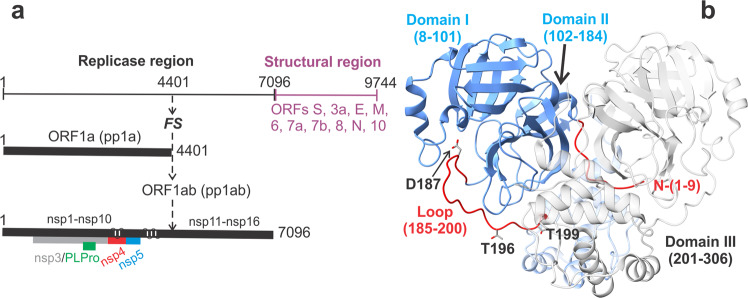
Genome organization of SARS-CoV-2: molecular representation and role of the main protease (MPro). **a** The ~30 kb genome codes for the various proteins in at least 12 open reading frames (ORFs). Two major polyproteins (pp) are encoded in ORFs, 1a (nsp1-nsp10) and 1ab (nsp1-nsp16), the processed proteins of which make up the replication/transcription complex. pp1ab is synthesized via a translation frameshifting (denoted FS) mechanism. The two virally encoded proteases PLPro (papain-like, green) and 3C-like main protease (MPro, blue) are responsible for the processing of pp1a and pp1ab. In the precursor form, MPro is anchored on either side with membrane spanning helices within nsp4 (red) and nsp6. MPro is responsible for its own release (termed self-cleavage or autoprocessing) and cleavage of the rest of the sites between nsp4 and nsp16. **b** Mature homodimeric MPro and regions critical for the modulation of the monomer-dimer (M-D) equilibrium. Subunits of the dimer are colored in blue and white. Regions defining the boundaries of domains are indicated. The loop region connecting the catalytic domain to the helical domain III (red) with D187, T196 and T199 residues shown as sticks. The free N-terminal strand, indispensable for inter- and intra-monomer interface contacts and dimer stability, is also shown in red just for the blue subunit.

**Fig. 2:**
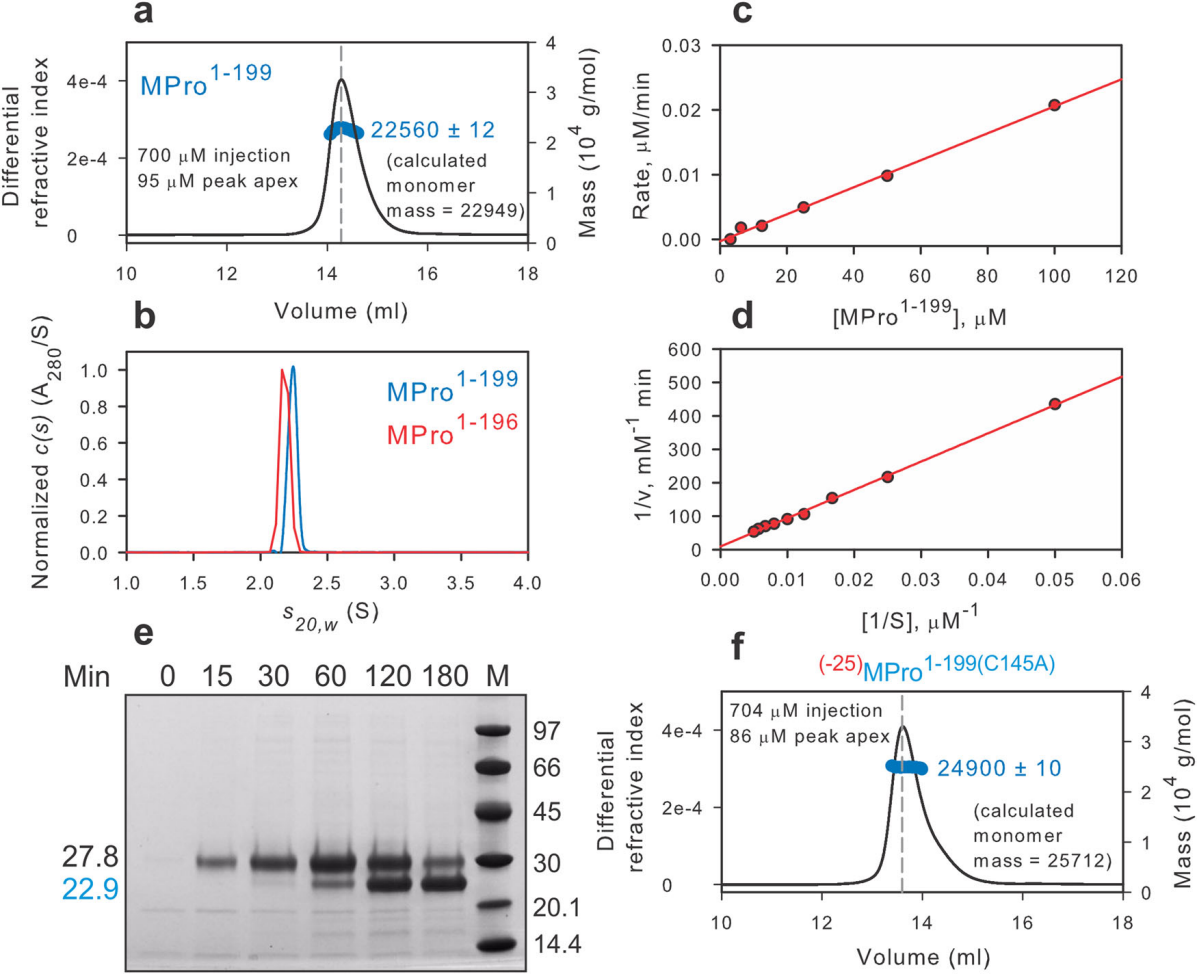
Molecular mass estimation and catalytic activity of monomeric MPro^1–199^ and its miniprecursor. **a** Molecular mass estimation of MPro^1–199^ by SEC-MALS. Fractionation was carried out as described in methods. The observed mass is indicated beside the peak. **b** SV-AUC absorbance *c(s)* distributions at loading concentrations of 201 and 184 µM of MPro^1–199^ and MPro^1–196^, respectively. **c** Linear relationship between the rate of catalyzed hydrolysis vs. the protein concentration of MPro^1–199^. **d** Lineweaver-Burk plot for hydrolysis of substrate by 90.5 µM MPro^1–199^. **e** N-terminal autoprocessing of the miniprecursor ^(−25)^MPro^1–199^ upon its expression in *E. coli*. The precursor, product released upon cleavage at the N-terminus of MPro and molecular weight standards (M) are indicated in kDa. **f** Molecular mass estimation of ^(−25)^MPro^1-199(C145A)^ by SEC-MALS. The observed mass is indicated beside the peak. Fractionation was performed as described in Methods.

The genomic organization of SARS-CoV-2 and a ribbon representation of the main protease are shown in Fig. 1. For the full-length monomeric double mutant (E290A, R298A) MPro^M^, the observed dissociation constant of the inhibitor GC373 (*K*_d_ = 1/*K*_a_ = *K*_i_ = 6.2 µM) and monomer-dimer equilibrium constant (*K*_dimer_) are identical^13^. To evaluate the binding of reversible covalent inhibitors to the active site of an exclusively monomeric form of MPro without the associated dimerization as observed for MPro^M^, a construct corresponding to residues 1–199 of MPro (MPro^1–199^) spanning the catalytic domain and the loop region was expressed and purified (Fig. S1). MPro^1–199^ elutes as a monomer at a peak apex concentration of 95 µM and an estimated mass of 22.6 kDa as shown by size-exclusion chromatography coupled to multi-angle light scattering (SEC-MALS) analysis (Fig. 2a). Consistent with this result, sedimentation velocity analytical ultracentrifugation (SV-AUC) of MPro^1–199^ at a loading concentration of 201 µM shows a species at 2.24S with an estimated mass of 21.7 kDa (Fig. 2b, blue trace). The distribution accounts for 99.4% of the sedimenting signal and has an integrated value of 0.963 absorbance units. A similar construct MPro^1–196^, lacking the C-terminal LE-6His residues, was also expressed and purified to provide a choice of two constructs for crystallization with and without the 6His-tag (Fig. S1). Like MPro^1–199^, MPro^1–196^ at a loading concentration of 184 µM also sediments as a monomer showing a species at 2.18S and an estimated mass of 20.5 kDa (Fig. 2b, red trace).

Similar to MPro^WT^ and MPro^M^, MPro^1–199^ catalyzes the hydrolysis of a known Förster resonance energy transfer (FRET) peptide substrate^3,13,33,34^ corresponding to the nsp4/nsp5 cleavage site in pp1a polyprotein^35^. Figure S2 shows chromatograms of the hydrolyzed FRET substrate by MPro^WT^ and MPro^1–199^, respectively. The catalytic activity with the FRET substrate is inhibited by the addition of NMV^11,12^. The rates of hydrolyses of the FRET substrate at a final substrate concentration of 200 μM displays a linear relationship with the concentration of MPro^1–199^ having an intercept at the origin [multiple correlation coefficient (*R* = 0.9984)] and calculated *k*_cat_/*K*_m_ is (1.0 ± 0.03)10^−6^ µM^−1^ min^−1^ (Fig. 2c and Table S1). The first-order dependency on the protein concentration indicates that the observed catalytic activity is that of a monomeric MPro^1–199^, and the protein is exclusively in the monomeric form. A plot of 1/v vs. 1/[S] at MPro^1–199^ concentration of 90.5 μM is linear (*R* = 0.9987) with a small positive intercept and the calculated *k*_cat_/*K*_m_ is [(1.3 ± 0.02)10^−6^ µM^−1^ min^−1^, Fig. 2d]. Thus, MPro^WT^ is 5.22 × 10^5^ times more active than MPro^1–199^, corresponding to 7.7 kcal/mol, representing the contribution of dimer formation to stabilizing the active form of the enzyme (Table S1).

To ascertain if MPro^1–199^ mediates its own cleavage as a polyprotein and further validate its intrinsic catalytic activity, MPro^1–199^ was expressed as a miniprecursor [^(−25)^MPro^1–199^] appended to 25 amino acids of its N-terminal flanking sequence which correspond to the C-terminal residues of nsp4 (Figs. 1a and S1). Therefore, the construct encompasses the nsp4/nsp5 junction sequence matching that of the FRET peptide substrate. Indeed, ^(−25)^MPro^1–199^ (27.8 kDa) promotes its own cleavage at the nsp4/nsp5 junction to produce products (22.9 and 4.9 kDa, Fig. 2e) in *E. coli* although at a slower rate than observed for MPro^WT^ (Fig. S3a) and MPro^M^ (Fig. S3b) also expressed as a precursor containing a truncated portion of its N-terminal flanking sequence. In agreement with the catalytic activity profile, MPro^WT^ >> MPro^M^ >>> MPro^1-199^ (Table S1), a similar progressive decrease in the rate of autoprocessing of these constructs is observed (Fig. S3, note that Fig. 2e is reproduced as Fig. S3c for ease of comparison). Consistent with MPro^1–199^ being a monomer, SEC-MALS analysis of a control construct ^+25^MPro^1–199(C145A)^ bearing an active site mutation, to restrict catalytic activity and enable the isolation and analysis of the intact precursor, showed a single eluting peak with an estimated mass of 24.9 kDa at a peak apex concentration of 86 µM (Fig. 2f). Like ^(−25)^MPro^1–199^, a miniprecursor construct ^(−25)^MPro^1–196^ which matches its processed counterpart MPro^1–196^ exhibits autoprocessing (Fig. S4a) validating its catalytic activity. However, a deletion to exclude the subsite 5 (S5, residues 189–194) loop sequence as in ^(−25)^MPro^1–187^ construct (Fig. S4b) results in only a very small fraction of the miniprecursor converting to products even after 3.5 h of induction, relative to ^(−25)^MPro^1–199^ and ^(−25)^MPro^1–196^ (compare Fig. S4b with S4a and Fig. S3c) indicating that subsite 5 is critical for catalytic activity.

### Inhibitor binding to monomeric MPro^1–199^, MPro^1–196^ and MPro^10–306^

A third construct MPro^10–306^ was included in this analysis in addition to MPro^1–199^ and MPro^1–196^. Construct MPro^10–306^ (Fig. S1) permits evaluation of the contribution of the N-terminal residues 1–9 influencing inhibitor binding in the presence of a full complement of the helical domain (residues 201–306, Fig. 1b). The binding constants of GC373 and NMV to these three constructs were determined by ITC. The *K*_d_s and the thermodynamic parameters are listed in Table 1 and compared to MPro^WT^ and MPro^M^^12,13^.

**Table 1:** Binding affinity of GC373 and NMV to MPro^WT^, MPro^1–199^, MPro^1–196^ and MPro^10–306^ as determined by ITC.

Compound	Chemical structure	Construct	*N*	*K*_d_ = *K*_i_ (µM)	Δ*H* (kcal/mol)	Δ*S* (cal/mol/K)	Δ*G* (kcal/mol)
GC373		MPro^WT^	0.99 ± 0.01	0.15 ± 0.03	−6.7 ± 0.1	9.1	−9.4
		MPro^M^	1.07 ± 0.02	6.13 ± 0.30	−6.0 ± 0.2	3.9	−7.2
		MPro^1–199^	0.9 ± 0.03	32 ± 5	−2.4 ± 0.1	12.7	−6.2
		MPro^1–196^	0.88 ± 0.09	45 ± 20	−1.48 ± 0.24	15	−6
		MPro^10–306^	0.54 ± 0.03	44 ± 8	−1.38 ± 0.1	15.3	−6
NMV		MPro^WT^	0.99 ± 0.003	0.007 ± 0.003	–10.75 ± 0.7	1.57	–11.2
		MPro^C145A^	0.96 ± 0.04	2.7 ± 0.9	–3.89 ± 0.2	12.6	−7.7
		MPro^1–199^	0.97 ± 0.04	19 ± 3	−3.9 ± 0.1	8.8	−6.5
		MPro^1–196^	1.05 ± 0.03	14 ± 3	−1.8 ± 0.1	16.3	−6.7

**Fig. 3:**
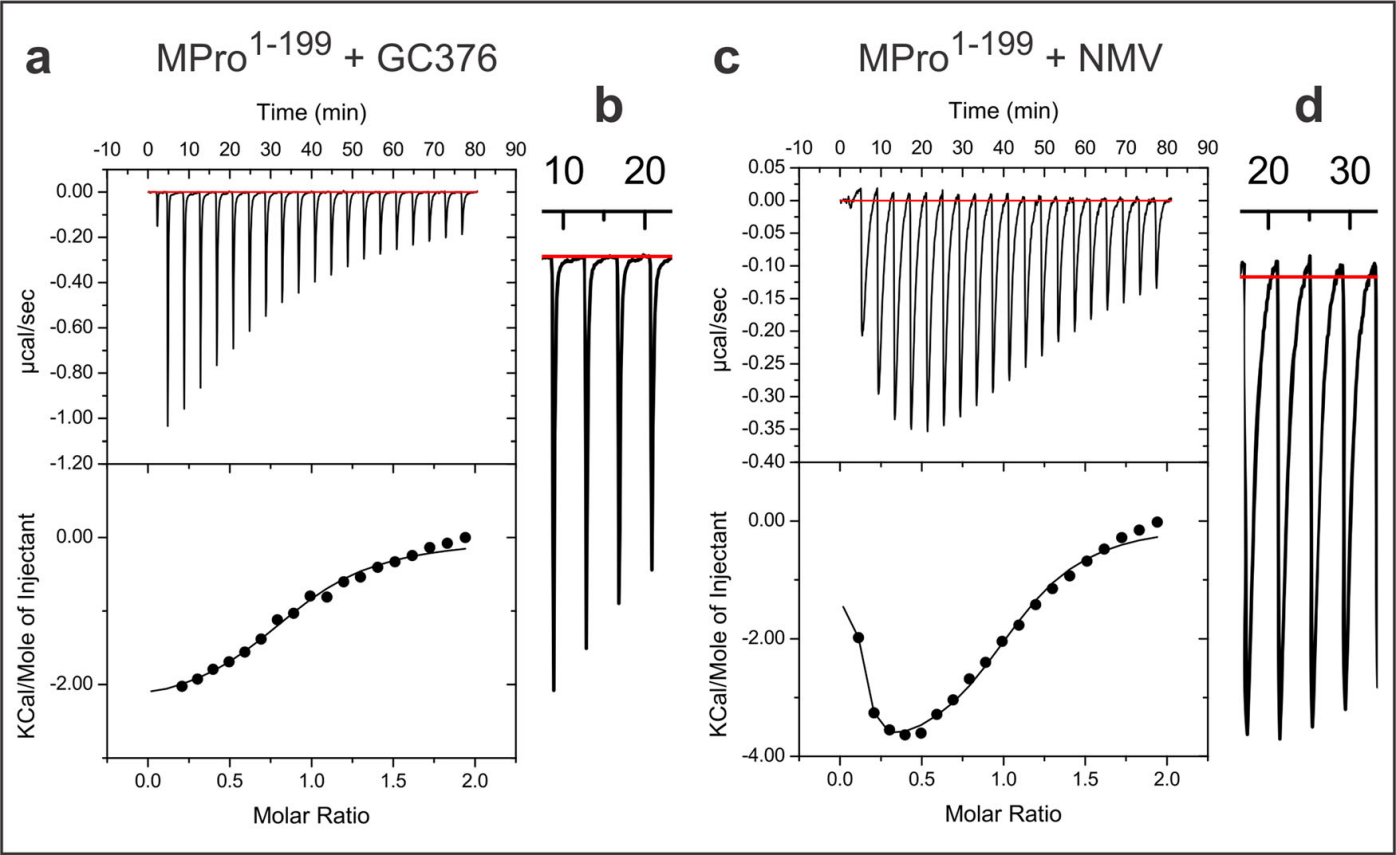
Binding isotherms of GC373 and NMV to MPro^1–199^. Titrations were carried out with MPro^1–199^ (in the cell) vs. **a** GC373 and **c** NMV (in the syringe) in buffer C at 28 °C. Enlarged view of a few deflections are shown for comparison when titrated with GC373 (**b**) and NMV (**d**). A slow thermal response is observed for the interaction of NMV with MPro^1–199^. This slow response is not observed when titrating NMV with MPro^WT^ (ref. ^12^, Fig. S7a) or MPro^C145A^ (this work, Fig. S7b). Thermodynamic parameters are listed in Table 1.

ITC experiments were carried out in buffer C at 28 °C. Data were processed and plots were generated with the Origin software provided with the instrument. Titrations of MPro^1–199^ with NMV shown in Figs. 3c and S6a were fit to a 2 sites model. The mean values obtained for the major isotherm are listed. Thermodynamic parameters derived for MPro^WT^ and MPro^M^ titrated with GC373, and MPro^WT^ titrated with NMV are cited from refs. ^12, 13^, respectively, solely for comparison with MPro analogs. No thermal response was observed when NMV was titrated with MPro^1–199^ (Fig. S6b) and MPro^10–306^ (Fig. S5d), and GC373 with MPro^C145A^ as listed in Table S1.

In an aqueous medium, the prodrug GC376 disproportionates to a sulfite ion and aldehyde GC373. GC373 inhibits MPro by reversibly binding and forming a covalent bond between the sulfur of C145 and the carbonyl carbon of GC373 to yield hemithioacetal^36,37^. Table 1 shows that *K*_d_ for GC373 increases from MPro^WT^ to MPro^10–306^. Raw heat deflections and binding isotherms are shown in Figs. 3 and S5. The *K*_d_ of GC373 to the catalytic domain alone and to MPro^10–306^ is 200 to 300-fold weaker compared to MPro^WT^ (*K*_d_ = 0.15 ± 0.03 µM^13^). The significant decrease in ΔH for binding, which accompanies weaker binding of GC373 to MPro^1–199^, MPro^1–196^ and MPro^10–306^ is offset by an increase in ΔS to give a net decrease in ΔG of about 3 kcal/mol. Notably, the values of Δ*G* are nearly the same for MPro^1–199^, MPro^1–196^ and MPro^10–306^, indicative of a common binding mode of GC373 and an active site conformational equilibrium that is similar in these constructs as compared to MPro^WT^ and MPro^M^. It is noteworthy that *K*_d_ decreases with increasing *k*_cat_/*K*_m_ (Table S1).

Similarly, NMV shows a weaker binding to MPro^1–199^ and MPro^1–196^ monomers, but with a 2000 to 2700-fold increase in the *K*_d_ relative to MPro^WT^ dimer (Table 1 and Figs. 3 and S5). The trend in the decrease in Δ*H* and increase in Δ*S* is also observed with a net Δ*G* difference of 4.5 to 4.7 kcal/mol (Table 1). Like GC373, the binding of NMV to MPro^1–199^ and MPro^1–196^ is a two-step process. The first step involves the formation of a noncovalent complex (fast step), followed by a slower step leading to the imidate thioester by forming a covalent bond between the sulfur atom of C145 and the nitrile carbon of NMV. Figure 3 shows the isotherms for binding of GC373 and NMV to MPro^1–199^. While the isotherm for binding of GC373 to MPro^1–199^ appears to display a single binding step with sharp heat deflections (Fig. 3a, b), the isotherm for NMV binding shows the two-step process with broader heat deflections (Fig. 3c, d), the latter being a dominant portion of the isotherm. The *K*_d_’s derived and listed in Table 1 correspond to the latter process and not the initial binding event. A duplicate titration with 30 injections shows a similar binding isotherm (Fig. S6a) and thermodynamic parameters.

The binding of NMV to MPro^1–199^ requires the presence of C145 as no thermal response was observed with MPro^1-199(C145A)^ (Fig. S6b). In contrast, the binding of NMV to MPro^WT^ is indicative of a fast reaction to the dimeric protein’s active site, with sharp heat deflections, which in part contributes to the higher affinity of NMV as shown before (Fig. S7a)^11,12^. While no thermal response was observed for the titration of NMV with MPro^10–306^ under our experimental conditions (Fig. S5d), reversed-phase liquid chromatography with in-line mass spectrometry (RPLC-MS) results indicated very little binding of NMV to the protein (see below). It is noteworthy that unlike NMV, GC373 binding to MPro^1–199^, MPro^1–196^ and MPro^10–306^ exhibit the typical binding with sharp deflections (Figs. 3a and S5a,c) like that observed when titrating with MPro^WT^^13^.

The results presented above indicate that NMV covalent complex formation requires the catalytically active conformation of the protein. The differences in binding of NMV to MPro^1–199^, MPro^1–196^ and MPro^10–306^ and those of GC373 may be attributed to the difference in the electrophilicity of the carbonyl carbon of GC373 and the nitrile carbon of NMV. Generally, the carbonyl carbon is more electrophilic and susceptible to nucleophilic attack than the nitrile carbon^38^, as oxygen is more electronegative than nitrogen. Also, the carbonyl group of the aldehyde GC373 is planar, mimicking the carbonyl of a peptide substrate. Thus, the carbonyl oxygen would be in a position to interact with the oxyanion hole of the protein and thereby enhancing the carbonyl carbon electrophilicity and the formation of the hemithioacetal. This interpretation is consistent with the binding of GC373 to MPro^10–306^ with a similar *K*_d_ and thermodynamic parameters as that with MPro^1–199^ and MPro^1–196^, although only half the protein is competent to bind to the inhibitor (*N* = 0.54, Table 1). In contrast, the nitrile group is linear, and the nitrile nitrogen may not readily interact with the oxyanion hole hydrogen bonds until the formation of the imidate thioester. This is evident from the absence of a thermal response in ITC for MPro^10–306^ with NMV (Fig. S5d) indicative of very little imidate thioester formation (see below).

### Oxyanion loop unwinding in monomeric MPro^1–199^

**Fig. 4:**
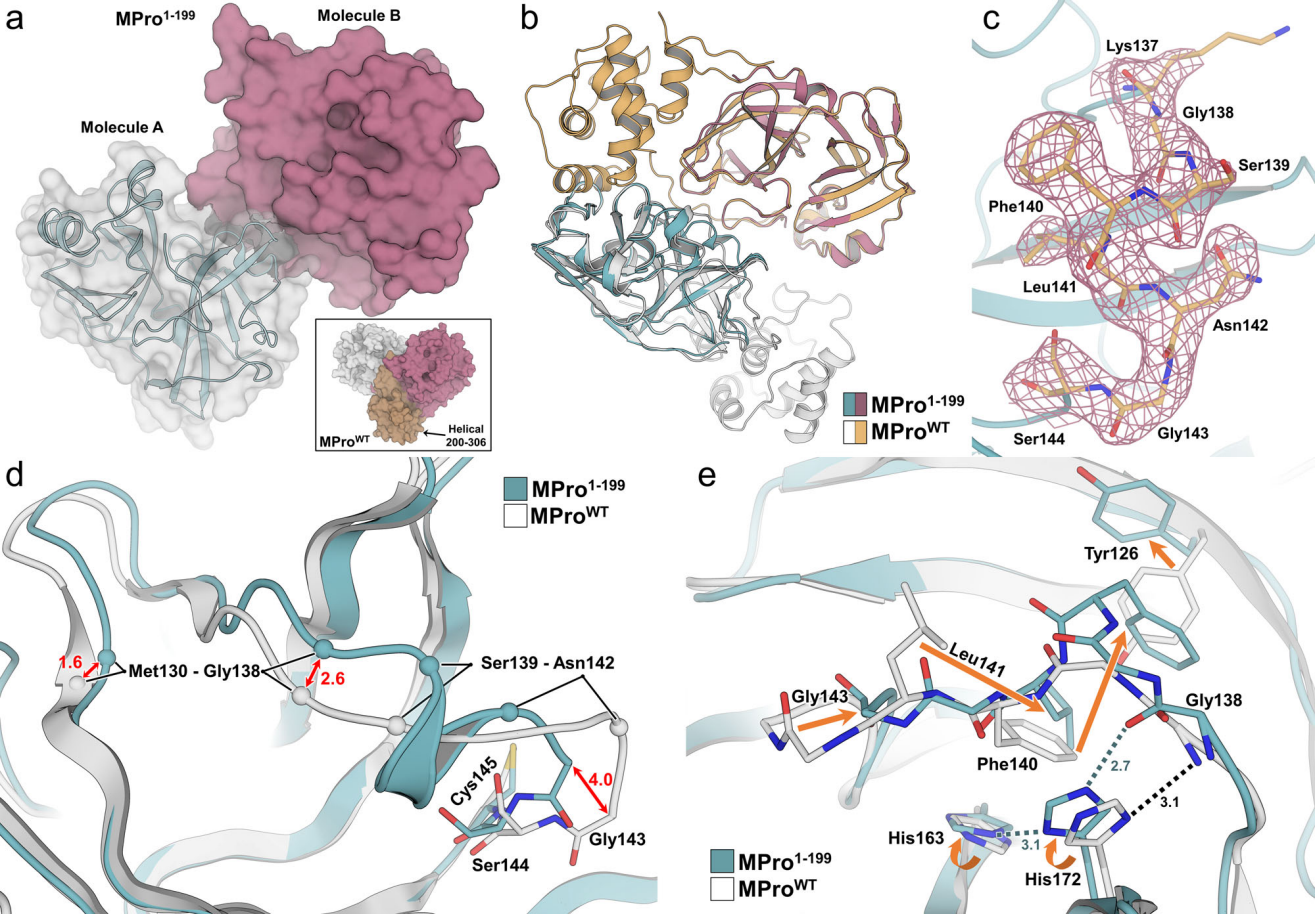
Room-temperature X-ray crystal structures of inhibitor-free MPro^1–199^ display unwinding of oxyanion loop. **a** MPro^1–199^ crystallized as two independent molecules in the asymmetric unit (molecule A: cyan cartoon with white surface, molecule B: mulberry surface). Inset shows MPro^WT^ (PDB ID 7JUN) crystallizes as one protomer per asymmetric unit with crystallographic symmetry generating the biologically active dimer assembly (helical domain from residues 201–306 shown as dark orange surface). **b** Superposition of MPro^WT^ to each independent MPro^1–199^ molecule indicates each MPro^1–199^ is a monomer that does not pack into the native dimer assembly. **c** Electron density of the oxyanion loop in MPro^1–199^ of molecule A (2Fo-Fc at 1σ, pink mesh). **d** Superposition of MPro^WT^ to MPro^1–199^ showing distance difference (Å) in protein backbone conformations as red arrows. In the absence of helical domain, Met130-Gly138 are in a different position and oxyanion loop residues 139–142 form a short helix. **e** Superposition of MPro^WT^ to MPro^1–199^ showing structural rearrangements of residues from positions in the native structure to the truncated structure as orange arrows. Hydrogen bonds for the flipped His172 sidechain are shown as dashed lines and all distances in Å. All superpositions performed as least-squares fit of Cα residues modeled in MPro^1–199^.

To understand the structural implications of our solution measurements of the enzymatic activity and inhibition of MPro^1–199^, we obtained its room-temperature structure in the inhibitor-free form at 2.25 Å resolution. MPro^1–199^ crystallizes with two independent molecules present in the asymmetric unit of P2_1_2_1_2_1_ space group that lacks a crystallographic 2-fold axis (Fig. 4a). Mature MPro^WT^ mainly crystallizes in space groups containing a crystallographic 2-fold axis so that the homodimer is formed through this symmetry operation^3,39,40^. This indicates that MPro^1–199^ is a monomer in the crystal. To provide further evidence MPro^1–199^ has a monomeric structure in the crystal, we superimposed both independent molecules with a monomer from the mature MPro^WT^ dimer^39^. As shown in Fig. 4b, the two independent molecules of MPro^1–199^ do not form a native dimer. These analyses demonstrate that MPro^1–199^ is indeed monomeric in the crystal as well as in solution. We modeled residues 7–188 in both independent molecules in MPro^1–199^, whereas the rest of the N-terminal and C-terminal residues are highly dynamic and are not visible in the electron density map. In MPro^1–199^, the electron density in the active site, specifically that of the oxyanion loop (residues 137–144, Fig. 4c), is stronger in molecule A, and the two crystallographically independent molecules superimpose with an RMSD of 0.4 Å. Therefore, the subsequent structure analysis is based on molecule A.

MPro^1–199^ superimposes onto the inhibitor-free MPro^WT^ (residues 7–188, PDB ID 7JUN) with the RSMD of 1.0 Å, which points to significant local conformational changes in the catalytic domain structure compared to the full-length mature enzyme (Fig. S8). Indeed, an unwinding of the oxyanion loop is evident, starting from Gly143 that shifts by >4 Å away from the catalytic Cys145, opening the substrate binding subsite S1 wider. This conformational shift squeezes the stretch of residues 139–142 into a short 3_10_ helix, which pulls a whole loop consisting of residues 130–138 closer to the newly formed helix by 1.2–1.6 Å (Fig. 4d). Residues 130–138 normally interact with the helical domain (residues 201–306), which is absent in MPro^1–199^. Therefore, their movement can also partially be due to the lack of the helical domain in the dissected enzyme. In the new configuration of the oxyanion loop, the side chain of Leu141 moves into the position previously occupied by the phenyl group of Phe140, whereas the latter shifts toward the Tyr126 phenolic group, forcing it to rotate by ~100° into the bulk solvent to avoid the clash (Fig. 4e). These structural rearrangements result in the imidazole side chain of His172 trading a hydrogen bond with the main chain amide NH of Gly138 in MPro^WT^ for a hydrogen bond (2.7 Å) with the main chain carbonyl of the same residue in MPro^1–199^. Moreover, the His172 imidazole’s Nδ1 is also within a hydrogen bonding distance of 3.1 Å to Nε2 of His163 in MPro^1–199^. Thus, it is possible that His172 becomes positively charged (i.e., doubly protonated) in MPro^1–199^, whereas it was found neutral (i.e., singly protonated) in MPro^WT^^39^, being locked in a hydrogen bond with Gly138. A similar unwinding of the oxyanion loop, including the formation of the 3_10_ helix described above, was previously observed in the full-length monomeric enzyme of SARS-CoV-1, MPro^R298A^, where a single R298A mutation caused dissociation of the mature homodimer^27^. The two structures, MPro^1–199^ and MPro^R298A^, superimpose with the RMSD on the main-chain atoms of 0.9 Å. The structural differences are born out mainly from the lack of the helical domain and disorder of the N-finger residues 1–6 in MPro^1–199^, because the majority of the atomic shifts are centered on the amino acid residues that interact with these portions of the enzyme. This includes residues 136–139 in the loop preceding the 3_10_ helix, residues 117–126 interacting with the N-terminus, and residues 7–12 following the N-finger. Interestingly, because MPro^R298A^ contains the helical domain, its N-finger (residues 1–7) is ordered but swung away from its position in MPro^WT^, making interactions with the helical domain residues such as Leu282 and Phe291. Moreover, the whole helical domain in MPro^R298A^ repositions itself by a 40° reorientation relative to the catalytic domain structure^27^.

The organization of substrate binding subsite S1 is preserved in MPro^1–199^ relative to its structure in MPro^WT^ even though the N-terminus of the second protomer that usually caps S1 as part of the mature homodimer is no longer present in the truncated enzyme. In the absence of the stabilizing effect of the N-terminus, the side chain of Glu166 rotates from its position observed in MPro^WT^, where it hydrogen bonds the N-terminus of the second protomer toward the oxyanion loop (Fig. S8). In the new conformation in MPro^1–199^, Glu166 makes a 3.0 Å hydrogen bond with the main chain amide NH of Gly143 that was part of the oxyanion hole in MPro^WT^. Of note, the S2 helix (residues 46-51) moves ~2 Å away from the catalytic site (Cys145/His41 dyad) and subsite S1. This movement results in the active site opening by ~1 Å in MPro^1–199^ relative to MPro^WT^ measured by the increase in the distance between Cα atoms of Ser46 in the S2 helix and Pro168 in the S4 β-hairpin loop (residues 165–170), a characteristic feature usually associated with binding of a ligand to the enzyme^10,12,33^. The active site widening may also in part be caused by the lack of the structured C-terminus in MPro^1–199^ compared to MPro^WT^, where residues 189–194 from the S5 loop directly interact with those in the S2 helix.

### Covalent inhibitors restore native active site conformation

To find out whether inhibitor binding to the truncated enzyme can restore the geometry of the oxyanion loop and the overall configuration of the active site to their native states, we obtained room-temperature X-ray crystal structures of MPro^1–196^ in complex with covalent inhibitors GC373 and NMV at 1.80 and 1.85 Å resolutions, respectively. We used the construct MPro^1–196^ to accelerate crystal growth because it lacks the 6His-tag^35^. However, crystallizing MPro^1–196^ in the absence of an inhibitor or MPro^1–199^ in the presence of inhibitors were unsuccessful. For structural comparisons, we also made a complex of full-length MPro^WT^ with GC373 (MPro^WT^-GC373) and obtained its room temperature X-ray structure at 2.0 Å resolution. In addition, we compare the MPro^1–196^-NMV complex with our recent 2.0 Å resolution structure of MPro^WT^-NMV (PDB ID 7SI9).

**Fig. 5:**
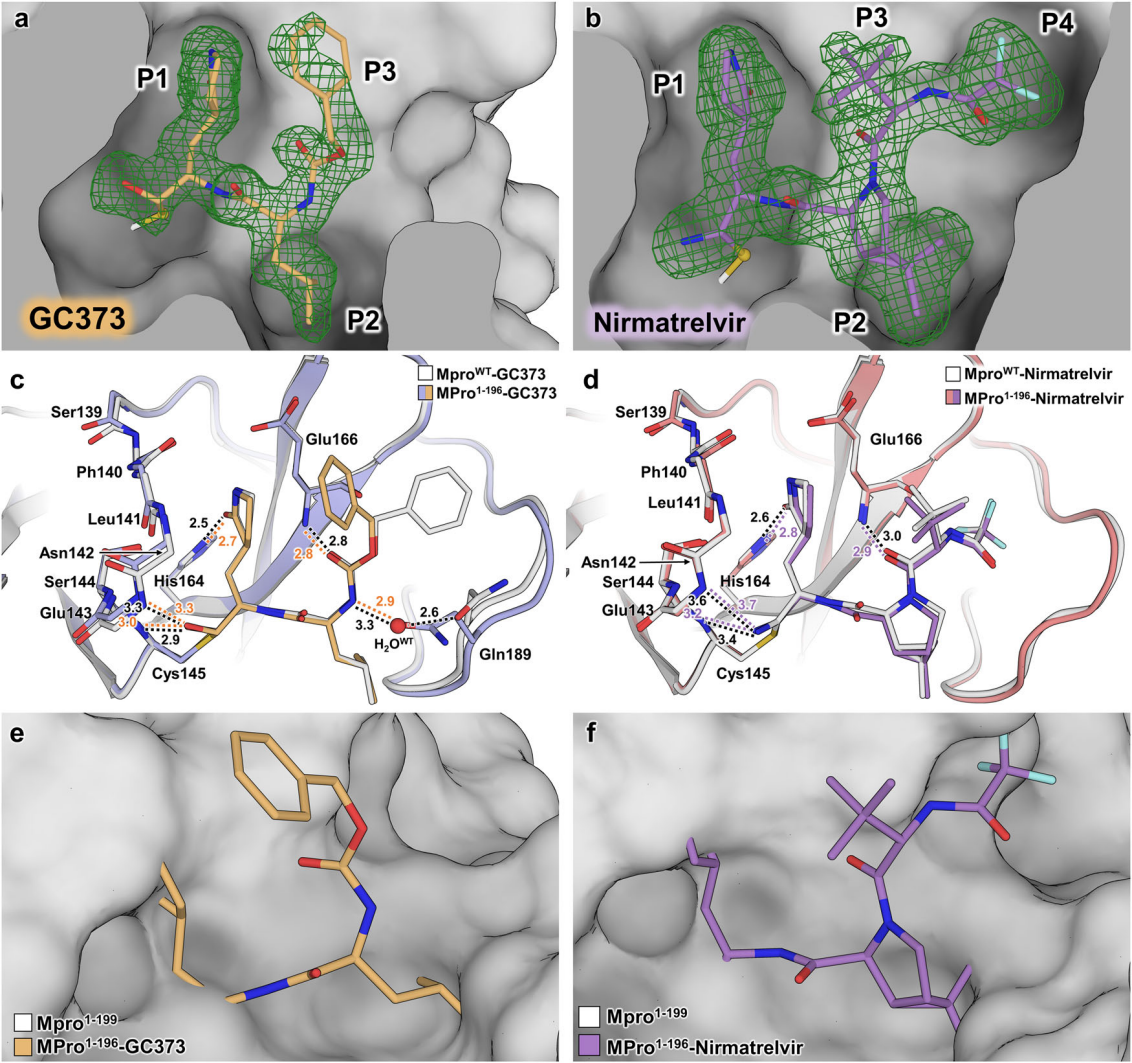
Room-temperature X-ray structures of MPro^1–196^ in complex with covalent inhibitors GC373 and NMV. **a**, **b** Electron density of GC373 (orange sticks) and NMV (purple sticks) in the active site of MPro^1–196^ molecule A shown as Polder omit maps (green mesh contoured at 3σ). **c**, **d** Superposition of MPro^WT^-inhibitor complex monomer with the equivalent MPro^1–196^-inhibitor complex (molecule B). The main chain of oxyanion loop residues 139–145 are shown creating the oxyanion hole and interacting with ligand. Hydrogen bonds represented as color-coded dashes with distances in Å. All distances are shown in Å. **e**, **f** Superposition of inhibitor-free MPro^1–199^ with the GC373 and NMV MPro^1–196^ complex, respectively. Inhibitor-free MPro^1–199^ represented as white surface where the conformational shifts described result in an occluded ligand-binding site. All superpositions performed as least-squares fit of Cα residues modeled in MPro^1–199^.

MPro^1–196^-GC373 and MPro^1–196^-NMV complexes crystallize in the isomorphic monoclinic unit cells with a P2_1_ space group. Similar to MPro^1–199^, there are two independent protein molecules in the asymmetric units of the inhibitor complexes. The inhibitors in both structures show clear electron densities as indicated in Fig. 5a, b. Superimposition of the independent molecules in MPro^1–196^-GC373 (and in MPro^1–196^-NMV) with a monomer from the mature MPro^WT^ dimer^39^ clearly shows that the native dimer is not formed by the juxtaposition of the two independent molecules in these crystal structures. Thus, in agreement with our solution measurements, GC373 and NMV can covalently bind to the monomeric MPro^1–196^. Moreover, due to inhibitor presence, the C-termini in the MPro^1–196^-inhibitor complexes are better defined in the electron density maps, so we were able to model up to Gly195 (molecule B) in MPro^1–196^-GC373 and up to Thr196 (molecule B) in MPro^1–196^-NMV, the entire length of the construct at the C-terminus. Another salient feature is residues 189–194 belonging to the S5 loop which become ordered in the inhibitor complexes even though both inhibitors lack P5 groups.

MPro^1–196^-GC373 and MPro^1–196^-NMV (molecules B) superimpose on the corresponding catalytic domains of MPro^WT^-GC373 and MPro^WT^-NMV with RMSDs of 0.4 Å for main chain atoms, suggesting the matching pairs of the structures are very similar. A close inspection of the oxyanion loops in the MPro^1–196^-GC373 and MPro^1–196^-NMV complexes reveals that residues 139–142 adopt the native conformation in both structures (Fig. 5c, d). Thus, covalent inhibitor binding reverts the oxyanion loop geometry to its active configuration. In MPro^1–196^-GC373, the oxygen atom of the inhibitor’s hemithioacetal conjugate makes a 3.0 Å hydrogen bond with the main chain amide NH of Cys145 and longer (3.4–3.5 Å) contacts with the main chains of Gly143 and Ser144. The corresponding interactions in MPro^WT^-GC373 are slightly, but not significantly, shorter, 2.9 Å with Cys145 and 3.3 Å both with Gly143 and Ser144. Therefore, the hydrogen bonding with the oxyanion hole is virtually identical in the two structures. A hydrogen bond formed by the carbonyl of the P1 γ-lactam with the His163 imidazole is also slightly shorter (2.5 Å) in MPro^WT^-GC373 than in MPro^1–196^-GC373 (2.7 Å).

Conversely, the carbonyl of the carbamate group makes identical 2.8 Å hydrogen bonds with the main chain amide NH of Glu166 in both structures, whereas carbamate’s NH recruits the side chain of Gln189 in MPro^1–196^-GC373 forming a direct 2.9 Å hydrogen bond. In MPro^WT^-GC373, Gln189 is instead rotated away from the inhibitor facing the bulk solvent, and a water molecule is inserted between the carbamate’s NH and Gln189 side chain, replacing a direct contact with a water-mediated interaction (Fig. 5c). Similar to GC373, NMV interacts identically with the MPro^1–196^ active site as it does with the native protein in the MPro^WT^-NMV complex (Fig. 5d). Direct hydrogen bond distances differ by only 0.1–0.2 Å in the two structures. Hence, the structural analysis demonstrates that binding of the covalent inhibitors to the monomeric enzyme leads to the conformational changes culminating in restoring the native structure of the active site and very similar enzyme-inhibitor interactions to those in MPro^WT^.

We also superimposed the inhibitor complexes MPro^1–196^-GC373 and MPro^1–196^-NMV onto the inhibitor-free structure of MPro^1–199^ to visualize the differences in the active site organization and conformation. As is evident from Fig. 5e, f, when MPro^1–199^ is represented with a surface, the inhibitor groups P1 and P2 clash with the MPro^1–199^ residues in subsites S1 and S2, providing further evidence that the active site shape in the inhibitor-free structure does not match the inhibitor structures. Thus, GC373 and NMV would either bind to the monomeric enzyme by induced fit, reshaping the active site into the active conformation, or bind through the conformational selection, when some proportion of MPro^1–196^ molecules contains active sites with the appropriate conformation for the inhibitor binding.

Our structures indicate that inhibitor bound conformation of the oxyanion loop of MPro^1–199^ is nearly identical to that of MPro^WT^. Thus, it is the transitioning of the oxyanion loop from the unwound to the native state that likely accounts for the weaker binding and measured differences in the thermodynamic parameters observed for MPro^1–199^ and MPro^1–196^, relative to MPro^WT^. Presumably, the Δ*H* is related to the magnitude of GC373 and NMV reactivity, and ΔS to the associated conformational changes including those of the terminal residues and the exclusion of the hydration water from the active site.

### Inhibitor binding promotes dimerization of MPro^1–199^ and MPro^1–196^

As stated above, the binding of GC373 to a predominantly monomeric MPro [MPro^M^^13^,] is accompanied by an increase in dimer formation and catalytic activity. To ascertain the influence of binding of GC373 and NMV on dimer formation in the absence of the helical domain or the N-terminal residues 1-9, a series of SV-AUC analyses were carried out at concentrations of ~50 and ~200 µM of MPro^1–199^, MPro^1–196^ and MPro^10–306^ and two-fold molar excess (hereafter also referred to as 2×) of inhibitors GC373 and NMV, the higher concentrations reflect nearly the same protein/inhibitor ratios upon completion of the ITC experiments (~200 µM).

**Fig. 6:**
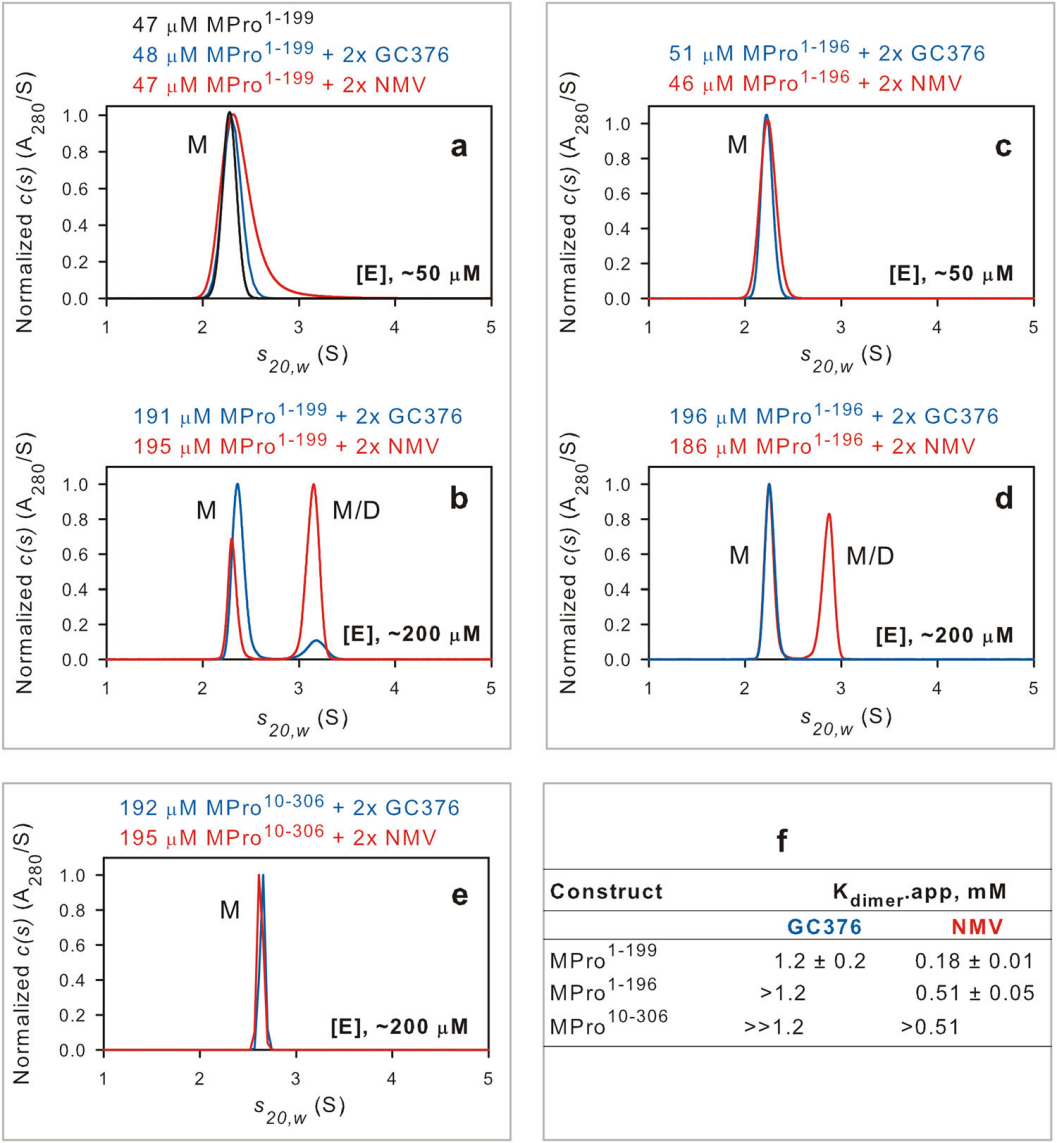
Estimation of the apparent *K*_dimer_ of MPro^1–199^, MPro^1–196^ and MPro^10–306^ in the presence of inhibitors. SV-AUC absorbance c(s) distribution at ~50 µM (**a**, **c**) and ~200 µM (**b**, **d**, **e**) loading concentrations in the presence two-fold molar excess (2x) of either GC373 or NMV as indicated. [E] denotes enzyme concentration. Protein concentrations used in **b**, **d**, **e** match those recovered after ITC. **f** Apparent dimer dissociation constants in the presence of 2x inhibitor. Actual concentrations are indicated above the plots. M and M/D denote monomer and monomer/dimer equilibrium boundary, respectively.

At a loading concentration of ~50 µM MPro^1–199^ and in the absence of GC373, a single species at 2.28S accounts for the total signal with an estimated mass of 21.6 kDa (Fig. 6a). This single peak slightly shifts to 2.31S and becomes slightly broader in the presence of 2x GC373 indicative of a small second population in fast equilibrium with the major monomeric species. A clear separation of two peaks corresponding to 2.34 (21 kDa) and 3.1 S (32 kDa) is observed with ~200 µM MPro^1–199^ with 2× GC373, the faster sedimenting peak accounting for about 14% of the signal (Fig. 6b). The same trend is observed in the presence of NMV mixed with MPro^1–199^, at ~50 µM the 2.38S peak gets even broader, and at ~200 µM, the 2.31S (19.7 kDa) and 3.14S (31.2 kDa) species account for 32% and 68% of the integrated signals (Fig. 6a, b).

Unlike MPro^1–199^, no dimer was observed at ~200 µM MPro^1–196^ concentration with 2× GC373, presumably due to the shortened loop region by three amino acids. The observed S values were 2.23 and 2.26 at ~50 and ~200 µM MPro^1–196^, respectively, in the presence of GC373 and no significant broadening of the peak is observed (Fig. 6c, d). In the presence of NMV and ~50 µM MPro^1–196^, a single peak corresponding to a monomer of 2.24S is observed with no significant broadening of the peak as seen with ~50 µM MPro^1–199^ mixed with NMV (Fig. 6c). At ~200 µM MPro^1–196^ mixed with 2× NMV, two species with equal distribution (50:50) corresponding to 2.25 and 2.86S were evident (Fig. 6d). It is of significance that at ~200 µM MPro^10–306^ mixed with 2× the concentration of either inhibitor, only sharp peaks of the monomer and no dimer peak was observed (Fig. 6e). The observed S values were 2.65 with an estimated mass of 33.8 kDa in the presence of 2x GC373 and 2.63 with an estimated mass of 34.6 with 2× NMV.

Interestingly, our ITC data indicates that GC373 is a better binder to monomeric MPro^10–306^, relative to NMV. It could be that the oxyanion loop equilibrium does not shift as readily to the inhibitor bound state with NMV in the absence of residues 5–9, this being yet another equilibrium process (or conformational selection) coupled to the oxyanion loop equilibrium.

Apparent dimer dissociation constants (*K*_dimer_.app) for ~190 µM MPro^1–199^ in the presence of GC373 or NMV, and 186 µM MPro^1–196^ in the presence of NMV were determined by single concentration Lamm equation modeling of the absorbance and interference sedimentation velocity data as described in the Experimental section. The best-fit dimer dissociation constants are listed in Fig. 6f (see Fig. S9). In addition, a side-by-side comparison of the estimated *K*_d_ and *K*_dimer_.app values by ITC and SV-AUC, respectively, and the fold difference *K*_dimer_.app/*K*_d_ are listed in Table S1. It is evident from this data that *K*_dimer_.app for MPro^1–199^ and its analogs in the presence of inhibitor is significantly higher than the corresponding *K*_d_, contrary to that observed for MPro^M^ previously, pointing to a distinct separation of inhibitor binding from dimer formation. As pointed out earlier, GC373 binding to the predominantly monomeric MPro^M^ leads to dimer formation concomitant with restoring the oxyanion loop conformation to the active state. Contrastingly, the results presented in this study indicate that inhibitor binding to monomeric MPro^1–196^ leads to establishing the active conformation of the oxyanion hole and the thermodynamic stability of the resulting complex promotes dimer formation.

### Identifying an adduct of NMV and not that of GC373 in solution

**Fig. 7:**
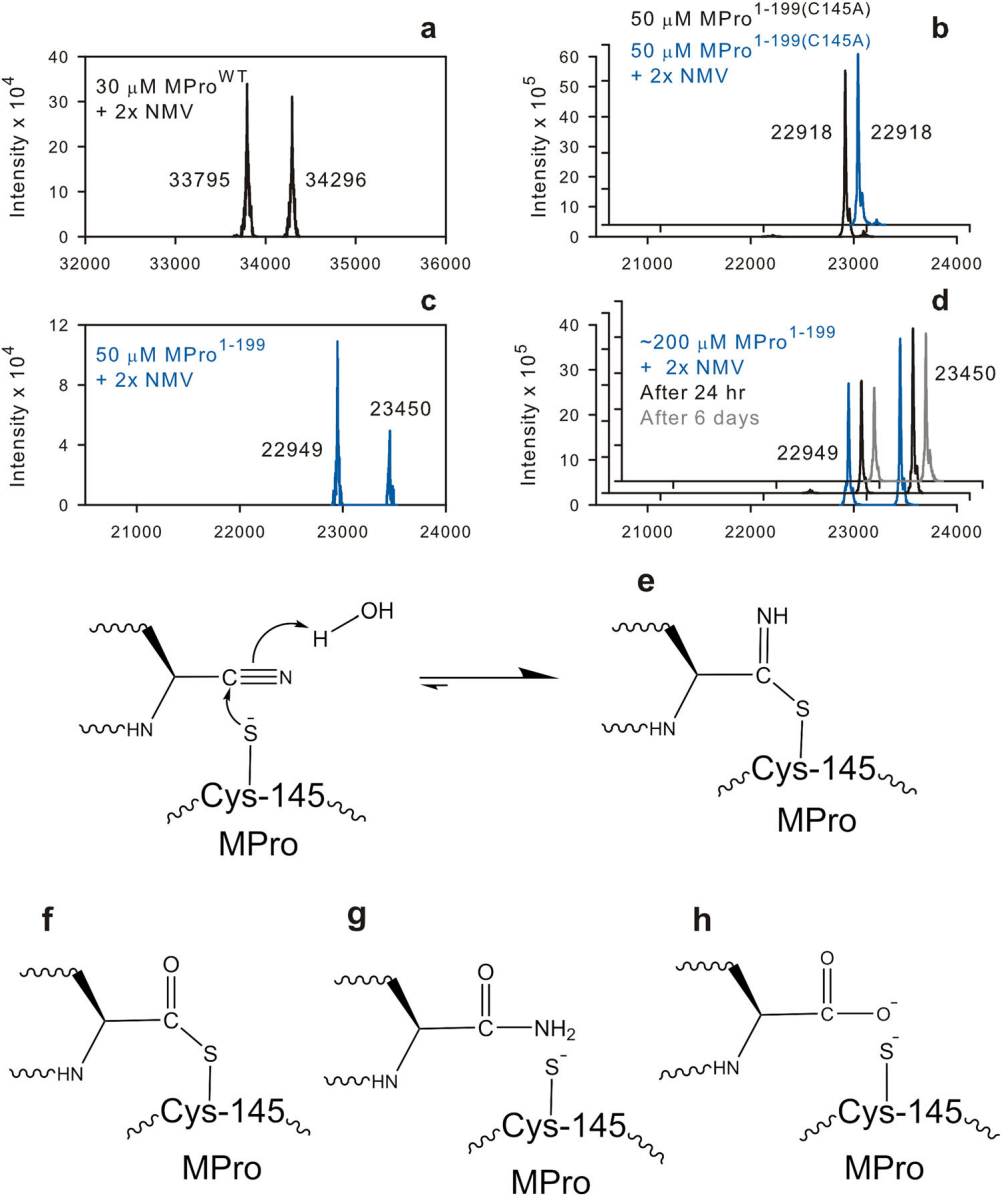
Identification of NMV adduct bound to MPro constructs. **a**, **c** Mass spectra of NMV adduct bound to MPro^WT^ and MPro^1–199^. **a**, **d** Samples recovered from the cell after ITC. Actual concentration of **d** is as shown for SV-AUC plot in Fig. 6b. Proteins were mixed with NMV at the indicated concentrations (**b**, **c**). Calculated masses are shown in Fig. S1 under each amino acid sequence of the corresponding construct. Black and gray traces in **d** indicate samples upon dilution to 20 µM and incubation for 24 h and 6 days, respectively, prior to subjecting 10 µl to RPLC-MS. **e**–**h** Mechanism of formation of NMV-MPro imidate thioester and possible products of its hydrolysis. **e** Imidate ester, **f** thioester, **g** amide, and **h** carboxylic acid.

The K_d_ for the binding of NMV to MPro^C145A^ is 2.7 µM as determined by ITC (Table 1 and Fig. S7). Thus, the nitrile warhead reacting with C145 increases the binding affinity by ~400 times, with a measured *K*_d_ of 7 nM for NMV with MPro^WT^. Since imidate thioesters are known compounds and significantly more stable than hemiacetals and hemithioacetals, proteins mixed with either GC373 or NMV and equilibrated for 2 h were analyzed by RPLC-MS. Typically, incubated mixtures of protein (50 and 200 µM) and 2× inhibitor, similar to those used in Fig. 6 for SV-AUC, were diluted to a concentration of 20 µM in 5% aqueous acetic acid and 10 µl of the sample was subjected to RPLC-MS. Fractionation by RPLC results in a dilution of the sample based on their retention volumes by 500 to 600-fold for MPro^1–199^ and MPro^1–196^ and ~700 fold for MPro^WT^ and MPro^10–306^. The mass spectra show a nearly equal distribution of MPro^WT^ and the MPro^WT^-NMV adduct (Fig. 7a). In 50 µM mixtures, representing a concentration ~3-fold above the observed dissociation constants (Table 1), protein-NMV adducts were observed for MPro^1–199^, MPro^1–196^, MPro^10–306^ (Figs. 7c and S10a, c). In a control experiment, only the molecular ion for MPro^1-199(C145A)^ was observed and not of the protein-adduct (Fig. 7b). This is consistent with the very weak or lack of binding observed with ITC (Fig. S6b) and no faster sedimenting species other than a monomer was observed (Fig. S6c). At higher concentrations (~200 µM) of protein-NMV mixtures, MPro^1–199^, MPro^1–196^ and MPro^10–306^ show an increased population of the adduct (Figs. 7d and S10b, d). The protein-adducts are stable for days when maintained at 20 µM in acetic acid at ambient temperature as shown in Figs. 7d and S10b. Even though ITC and SV-AUC data suggest either very weak or lack of binding of NMV and its associated dimerization with MPro^10–306^, as seen for MPro^1–199^ and MPro^1–196^, mass spectra indicate that NMV binds to the active site of MPro^10–306^ as it does with MPro^WT^. This indicates the presence of a small population of MPro^10–306^ having the appropriate conformation to allow NMV binding that leads to the formation of the imidate thioester adduct. In contrast to the observed adducts with NMV, mass spectra of mixtures containing GC373 display only the molecular ions of the unmodified protein as verified both with MPro^1–199^ and MPro^1–196^ incubated at high concentrations and subjected to RPLC-MS.

The adduct is an imidate thioester (Fig. 7e) observed with MPro^WT^ and MPro^1–196^ as shown in crystal structures reported previously^11,12^. Also, GC373 is known to form a covalent hemithioacetal adduct to the catalytic C145 of MPro^WT^ which we have not observed in RPLC-MS analysis. There are two possible chemical pathways for the decomposition of the adduct in aqueous solution. As suggested by Owen et al.^11^, the formation of the imidate thioester is reversible, but the reverse reaction is slow, leading to its accumulation in solution. Alternatively, imidate thioesters are reactive esters susceptible to hydrolytic reactions leading to the formation of amides and/or thioesters^41^ which may be subject to further hydrolysis to the corresponding carboxylic acids. Proteases such as α-chymotrypsin, subtilisin and carboxy-peptidase. A have been reported to catalyze the hydrolyses of ester and amide substrates in which the ester or amide carbonyl oxygen is replaced with a sulfur, nevertheless at much slower rates than the corresponding amides and esters^42,43^ and thus, it is possible for MPro^WT^ to catalyze the hydrolysis of the imidate thioester adduct (Fig. 7e) to produce one or more of the three possible products: thioester, amide, and carboxylic acid (Fig. 7f–h). It should be noted that thioesters and amides are substrates that can undergo catalytic hydrolysis by the enzyme to produce the carboxylic acid as an end product. Our results and the conclusion presented above are consistent with the results obtained for the inhibition of cysteine proteases such as papain by nitrile analogs of their substrate which establish the formation of imidate thioester adducts via an enzymatic process^44–46^. Imidate thioester of papain was reported to undergo hydrolysis to carboxylic acid and free enzyme in a very slow process^45^.

### Conformational stabilization of the oxyanion loop upon inhibitor binding promotes dimer formation

**Fig. 8:**
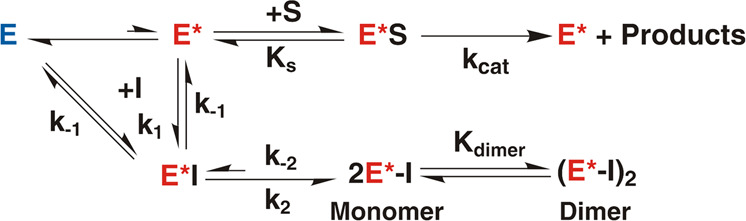
Inhibitor-binding induced conformational change of the oxyanion loop and dimerization of MPro^1–199^ and its analogs. E, E* and I denote active site conformation of a monomer (E, inactive state) which is in equilibrium with an active state (E*) resembling the active dimer, and inhibitor, respectively. Inhibitor bound active states before (E*I) and after (E*-I) covalent bond formation. K_−2_ is slow for NMV because of adduct formation in solution and not for GC373 for which no adduct could be observed by RPLC-MS.

The results listed in Tables 1 and S1 indicate that inhibitors GC373 and NMV bind to monomeric MPro^1–199^ (and MPro^1–196^) with *K*_d_’s in the range of 32–45 µM and 14–19 µM, respectively. In contrast, the apparent *K*_dimer_s are more than 175 µM. The observed catalytic activity of MPro^1–199^ indicates the presence of a very small fraction being in the catalytically competent conformation (E*) resembling the active site of the MPro^WT^ dimer. The scheme in Fig. 8 is a proposed mechanism to account for the observed results with NMV. Both MPro^1–199^ and MPro^1–196^ are mostly in the inactive conformation (E). Binding of GC373 or NMV to MPro^1–199^ and MPro^1–196^ leads to a rearrangement of the oxyanion loop equilibrium (E–E*) to that of the active form (E*) of the enzyme confirmed by our X-ray structures. Such an E → E* rearrangement and accompanied conformational changes facilitate dimer formation. The *K*_dimer_.app for dimer formation of MPro^1-199^*GC373 complex is 38-fold greater than the dissociation constant (*K*_d_ = 32 ± 5 µM) of GC373 to the monomeric MPro^1–199^. Similarly, *K*_dimer_.app for dimer formation of MPro^1-199^*NMV complex is 9-fold greater than the dissociation constant (*K*_d_ = 19 ± 3 µM) of NMV to the monomeric MPro^1–199^ (summarized in Table S1). This difference is 36-fold for MPro^1–196^. Lack of discernable dimerization of MPro^10–306^ in the presence of inhibitors up to ~200 µM suggests that the N-terminal residues 5–9, for which density is accounted for in the inhibitor bound crystal structures of MPro^1–199^ and MPro^1–196^, play a critical role in stabilizing the catalytically active conformations of E* and/or E*I of the protein, the latter being more predisposed to dimer formation.

## Conclusions

The structural studies presented above indicate that the catalytic domain encompassing the region 1–199 of MPro adopts a native-like fold for residues 7–188 with an unwound oxyanion loop conformation (E), which defines the catalytically inactive state of monomeric MPro. The observed first-order dependency of the rate of hydrolysis by the catalytic domain on the protein concentration indicates that the monomeric catalytic domain exists in at least two conformers. A major inactive conformer (E) which is in dynamic equilibrium with an enzymatically active minor conformer (E*) having an active site oxyanion hole similar in conformation to that of the MPro^WT^ dimer. Covalent inhibitors GC373 and NMV bind to the monomeric catalytic domain, as shown from our crystal structures and ITC data. Structural studies of the catalytic domain in complex with GC373 and NMV show the native E*I conformation as well as the appearance of electron density for the S5 loop up to residue 196. The E*-NMV complex is predisposed to dimerize at a lower protein and inhibitor concentration because of its enhanced conformational stability, relative to the E*-GC373 complex. While NMV binds to MPro^1–199^ displaying a well-organized structure for the N-terminal residues 5–9 as well as facilitate dimer formation, NMV binds very weakly to MPro^10–306^ with no detectable dimer formation. This result confirms that in addition to residues 5–9, interactions of domain III with residues 1–4 further enhance dimer stability.

The observed autoprocessing at the N-terminus of the miniprecursor ^(−25)^ MPro^1–199^ to produce the catalytic domain MPro^1–199^ indicates that both, the catalytic domain, and its miniprecursor are enzymatically active exhibiting an E-E* equilibrium. Deletion of the S5 loop (residues 189–194) drastically impairs the N-terminal autoprocessing. Based on the ability of substrates and inhibitors to promote an equilibrium shift to the E* form^13,30,31,47^, we propose that autoprocessing of MPro from its precursor polyprotein may also be governed by the binding of the N-terminal cleavage site sequence (nsp4/nsp5). This binding stabilizes the conformation of the oxyanion loop in the E* conformation leading to the liberation of its own free N-terminus. Thus, the conformational stability of the mature dimer is a collective effect of several interactions such as the interface formed between the N-terminal residues 1–9 with the reoriented domain III as well as the capping of the S1 subsite through the interaction of S1 residue of one subunit with F140/E166 of the second subunit when present in an active E* loop conformation. The E state of monomeric MPro or its polyprotein precursor present a strategic target for inhibitor design.

## Methods

### Construction and designation of MPro constructs

The expression and purification of MPro^WT^ (GenBank ID: MN908947.3) were carried out as described^10,12,13^. All constructs were synthesized and cloned into pJ414 vector (ATUM, Newark, CA). MPro^1–199^ consists of the catalytic domain (residues 1–184) followed by the loop region (residues 185–199) and a C-terminal LE-6His. The same construct but with a C145A mutation is termed MPro^1–199(C145A)^. MPro^1–196^ spans residues 1–196, but flanked by 6His, spacer and TEV protease cleavage site at the N-terminus of the MPro sequence to facilitate its purification without any non-native residues upon TEV protease cleavage. MPro^10–306^ spans the region 10–306 of MPro followed by GP-6His to facilitate its purification followed by removal of GP-6His via human rhinovirus 3C (HRV-3C) protease cleavage. Construct ^(−25)^MPro^1–199^, a miniprecursor mimetic of MPro, encompasses residues 1–199 of MPro fused to 25 amino acids of the native flanking C-terminal residues of nsp4. This construct contains a 6His at both ends as shown in Fig. S1. A similar construct ^(−25)^MPro^1–199^ bearing an active site C145A mutation but with only the 6His at the C-terminus was also synthesized and cloned. Construct ^(−25)^MPro^1–196^ is similar to ^(−25)^MPro^1–199^ with a 6His-tag only at its N-terminus. Construct^(−25)^MPro^1–187^ spans residues 1–187 of MPro fused to 25 amino acids of the native flanking C-terminal residues of nsp4. Amino acid sequence and designations of all MPro constructs used in this study are listed in Fig. S1.

### Expression and purification

Plasmids were transformed into BL21-DE3 cells (Agilent) and induced for expression at 0.7–0.8 optical density with 1 mM isopropyl β-d-1-thiogalactopyranoside typically for 3 h. Proteins were purified from the cell lysate by nickel-affinity chromatography (NAC, step 1). The bound fraction was subjected to isocratic fractionation on Superose-12 column (step 2, Cytiva Life Sciences) and HRV-3C protease cleavage (step 3, purchased from Sigma-Aldrich) or TEV protease (produced in-house^48^,) overnight at 4 °C followed by repeating NAC and step 2 in a final buffer of 25 mM Tris-HCl, pH 7 or 7.6, 150 mM NaCl and 1 mM TCEP (buffer A). The full-length wild-type (MPro^WT^) was expressed and purified similar in strategy to that described previously except for substituting the fusion partner GST with maltose binding protein (MBP) followed by a 36 amino acid spacer sequence corresponding to the immunoglobulin binding domain B1 of protein G (ΔGB1). Peak fractions were pooled and concentrated to the desired concentration and stored in aliquots at −30 °C and for long term storage at −80 °C. Purity was verified both by SDS-PAGE on 4–20% gradient mini-protean TGX precast gel (Bio-Rad) and electrospray ionization mass spectrometry.

### Size exclusion chromatography with multi-angle light scattering (SEC-MALS)

Molecular mass of MPro^1–199^ and ^+25^MPro^1–199(C145A)^ was estimated by analytical SEC with in-line MALS (DAWN Heleos-II, Wyatt Technology Inc., Santa Barbara, CA), refractive index (Optilab T-rEX, Wyatt Technology Inc.) and UV (Waters 2487, Waters Corporation, Milford, MA) detectors. Sample (125 µl) was applied onto a pre-equilibrated Superose-12 column (1.0 × 30 cm, Cytiva) and eluted at a flow rate of 0.5 ml/min in buffer A at 25 °C. Molecular mass was calculated using the Astra software provided with the instrument.

### Sedimentation velocity analytical ultracentrifugation (SV-AUC)

Various constructs at protein concentrations of ~50 and ~200 µM in the absence and presence of 2-fold molar excess inhibitor were subjected to SV-AUC in buffer B (25 mM Tris-HCl, pH 7, 50 mM NaCl and 1 mM TCEP) and C (25 mM Tris-HCl, pH 7.2, 20 mM NaCl and 1 mM TCEP), respectively. Samples containing the inhibitor were prepared using a 1–4 mM stock solution of GC373 or NMV in buffer C to achieve the desired protein and inhibitor ratios and incubated for a period of 1–2 h prior to filling the cells.

Sedimentation velocity experiments were conducted at 50,000 rpm and 25 °C on a Beckman Coulter ProteomeLab XL-I analytical ultracentrifuge following standard protocols^49^. Samples were loaded in 2-channel centerpiece cells and scans were collected using both the absorbance (280 nm) and Rayleigh interference (655 nm) optical detection systems. Sedimentation data were time-corrected and analyzed in SEDFIT 16.1C^50^ in terms of a continuous c(*s*) distribution of Lamm equation solutions. Solution densities ρ, solution viscosities η, and protein partial specific volumes were calculated in SEDNTERP^51^. To obtain estimates of the dimer dissociation constants absorbance and interference sedimentation velocity data collected at a single concentration were analyzed globally using Lamm equation modeling in SEDPHAT 15.2b^52^. A monomer-dimer self-association model was used and the presence of both monomer and dimer species was confirmed in the analysis. Absorbance extinction coefficients and interference signal increments used in the analyses were based on the amino acid composition, and calculated in SEDNTERP and SEDFIT, respectively. Data were plotted in GUSSI^53^.

### Enzyme kinetics

Activity assays using the FRET substrate Dabsyl-KTSAVLQ/SGFRKM-E(Edans)-NH_2_^3,13^, where (/) denotes the scissile peptide bond, were carried out in a total volume of 100 µl in buffer B (25 mM Tris-HCl, pH 7, 50 mM NaCl and 1 mM TCEP) at 28 °C. For details, see references^3,13,54^. The substrate was custom synthesized (Biomatik, Ontario, Canada), and GC376 and nirmatrelvir (NMV or PF-07321332) were purchased from Selleckchem, Houston, TX and MedChemExpress, Monmouth Junction, NJ.

### Time course of the autoprocessing reaction

After induction, 12 ml of culture was drawn at various time points as indicated, optical density was measured, chilled in ice briefly and harvested immediately at 4 °C and frozen. Small scale nickel-affinity chromatography (NAC) was performed by lysis in 700 µl of 25 mM Tris-HCl (pH 8), 150 mM NaCl, 20 mM imidazole and 2 mM 2-mercaptoethanol. The lysate was spun at full speed in an Eppendorf centrifuge. Derived supernatant was subjected to NAC according to instructions provided for using His SpinTrap (Cytiva) and eluted in 200 µl of buffer containing 500 µM imidazole. Protein concentration was estimated both by Bradford’s assay as well as absorbance at 280 nm. Equal volumes were subjected to SDS-PAGE, stained and imaged. Band intensities were quantified using ImageJ software when needed.

### Isothermal titration calorimetry (ITC)

Purified proteins were diluted from a stock solution to slightly above the desired concentration and dialyzed extensively against buffer C (25 mM Tris-HCl, pH 7.2, 20 mM NaCl and 1 mM TCEP). Concentrations were estimated after dialysis based on their 280 nm absorbance at least twice. Stock solutions of inhibitors in buffer C were diluted in the same buffer to the desired concentration. Titrations (20 or 30 injections) were performed with proteins (245 to 300 µM) kept in the cell and inhibitors at 10-times the concentration of the protein in the syringe at 28 °C on iTC200 microcalorimeter (Malvern Instruments Inc., Westborough, MA). Data were processed using the Origin software provided with the instrument. For competitive inhibitors that bind at only one site, the dissociation constant (*K*_d_ = 1/*K*_a_) is equivalent to the inhibition constant measured by enzyme kinetics (*K*_i_).

### Protein crystallization and room-temperature X-ray crystallography

MPro^1–199^, MPro^1–196^ and MPro^WT^ protein samples were concentrated to 5–8 mg/ml. Stocks of inhibitors were prepared at 50 mM NMV in 100% dimethyl sulfoxide (DMSO) and 10 mM GC373 in buffer C for crystallization purposes and stored at −30 °C. For co-crystallization, MPro^1–196^ was mixed with GC373 or NMV, and MPro^WT^ with GC373, at 1:3 molar ratio and allowed to incubate at room temperature for a minimum of 60 min before setting up crystal trays. Crystals were grown by sitting drop vapor diffusion methodology with 18–21% PEG3350, 0.1 M Bis-Tris pH 6.5 or pH 7.0 (1 ml) as the precipitant solution. Crystallization drops of 20 µl at 1:1 ratio were seed struck using the crystals of the native MPro in complex with noncovalent ligand Mcule-5948770040 as described^10,12^. Crystals of MPro^1–199^ appeared after several weeks and grew to the final size in about 2 months at 10 °C. Co-crystals of MPro^1–196^ in complex with GC-376 and NMV grew in several days of incubation at 14 °C. Crystals were mounted in MiTeGen (Ithaca, NY) room-temperature capillary setups for data collection.

All room temperature X-ray crystallographic data were collected with a Rigaku HighFlux HomeLab instrument equipped with a MicroMax-007 HF X-ray generator, Osmic VariMax optics, and a DECTRIS Eiger R 4 M hybrid photon counting detector. X-ray diffraction data were integrated using the CrysAlis Pro software suite (Rigaku Inc., The Woodlands, TX) then reduced and scaled using Aimless^55^ from the CCP4 suite^56^. Structures were solved by molecular replacement using Phaser^57^. PDB code 2QCY^27^ was used as a search model to solve the inhibitor-free structure MPro^1–199^ by first truncating the sequence in the model up to residue 6 at the N-terminus and down to residue 188 at the C-terminus. MPro^1–199^-GC373 and MPro^1–199^-NMV structures were solved in a similar fashion using PDB code 6XQU^33^. A full-length model from PDB 6XQU was used to solve the structure of MPro^WT^-GC373. Each model was iteratively refined with Phenix.refine from the PHENIX suite^58^ and COOT^59^. Geometry validation was aided by Molprobity^60^. All ligand restraints were generated with eLBOW^61^ using geometry optimized by quantum mechanical calculations in Gaussian 16 at B3LYP/6-31 g(d,p) level of theory^62^. Final data collection and refinement statistics can be found in Table 2.

**Table 2:** Crystallographic data collection and refinement statistics.

	MPro^1–199^ PDB ID 7UJ9	MPro^1–196^-GC373 PDB ID 7UJG	MPro^1–196^-NMV PDB ID 7UJU	MPro^WT^-GC373 PDB ID 7UKK
Data collection:	X-ray (in-house)			
Diffractometer	Rigaku HighFlux, Eiger R 4M			
Space group	P2_1_2_1_2_1_	P2_1_	P2_1_	I2
Wavelength (Å)	1.5406	1.5406	1.5406	1.5406
Cell dimensions:				
* a*, *b*, *c* (Å)	53.34, 63.09, 121.35	52.79, 61.93, 58.94	52.73, 62.61, 58.96	45.80, 53.54, 113.17
α, β, γ (°)	90, 90, 90	90, 97.8, 90	90, 99.1, 90	90, 100.5, 90
Resolution (Å)	121.35–2.25 (2.33–2.25)	61.93–1.80 (1.87–1.80)	62.60–1.85 (1.92–1.85)	55.63–2.00 (2.07–2.00)
No. reflections unique	20,094 (2012)	33,340 (3207)	32,399 (3115)	18,348 (1840)
*R*_merge_	0.137 (0.827)	0.038 (0.212)	0.072 (0.490)	0.071 (0.375)
*CC*_1/2_	0.952 (0.798)	0.996 (0.945)	0.997 (0.650)	0.994 (0.854)
*<I*/σ*I*>	11.05 (0.99)	32.30 (5.42)	15.32 (2.09)	19.24 (2.85)
Completeness (%)	99.7 (99.6)	95.1 (91.5)	99.3 (95.7)	99.9 (99.9)
Redundancy	7.0 (7.1)	4.8 (3.4)	5.1 (3.5)	5.0 (4.8)
Refinement:				
*R*_work_ / *R*_free_	0.2043/0.2502	0.1517/0.1821	0.1716/0.2067	0.1609/0.1814
*B*-factors (Å^2^)				
Protein	52.31	23.11	28.05	30.24
Ligand	N/A	24.20	27.67	28.84
Water	43.71	34.20	37.95	35.63
R.M.S. deviations				
Bond lengths (Å)	0.004	0.007	0.009	0.003
Bond angles (°)	0.677	0.958	1.090	0.560
All atom clash score	4.84	2.21	2.87	1.88

Data reduction and refinement statistics for the room temperature X-ray crystal structures of SARS-CoV-2 MPro^1–199^ and MPro^1–196^ in complex with GC373 and NMV, respectively, and MPro^WT^ in complex with GC-373. Values in parentheses are for the highest-resolution shell.

### Reversed-phase liquid chromatography-mass spectrometry (RPLC-MS)

Proteins and their inhibitor complexes were subjected to mass spectrometry, using the Thermo Dionex Ultimate 3000 HPLC system and Thermo MSQ Plus single quadrupole mass spectrometer^63^. Typically, 10 µl of sample at 20 µM concentration, diluted from a reaction mixture in 5% acetic acid, was loaded onto an Acclaim PepMap 300 C4 column (1.0 × 15 cm, Thermo Fisher Scientific) at 40 °C, with 0.2 ml/min flow rates in 2% acetonitrile/95% water/0.01% TFA. The column was washed for 10 min and the bound protein was fractionated using a linear solvent gradient from 2% acetonitrile/95% water/0.01% TFA to 60% acetonitrile/40% water/0.01% TFA over 25 min. Chromeleon software provided with the instrument and MagTran (Amgen) were used to analyze the data and estimate mass.

### Statistics and reproducibility

Expressed proteins were verified both by DNA sequencing and mass spectrometry. The reproducibility of enzyme kinetics was tested at least 2–3 times with freshly prepared enzyme and stock solutions of the substrate and inhibitor. Once this was determined to provide consistent reaction rates within an error limit of 5%, the final experiment for the data displayed in the manuscript was carried out in duplicate and 4 reads per well for each time point. The mean of the data points was used for fitting. The same stock solutions of enzyme and inhibitor were used for SV-AUC and ITC analyses to determine the dimer dissociation constant (*K*_dimer_) and the binding constant of the inhibitor to the enzyme (*K*_d_), respectively. *K*_dimer_, *K*_d_ and molecular mass were determined with multiple protein constructs (Fig. S1) and concentrations. Each ITC experiment was carried out with a minimum of 20 injections. The apparent dimer dissociation constants were determined by Lamm equation modeling of the absorbance and interference data. X-ray diffraction data and refinement statistics are shown. Gel images are best representative for each of the construct analyzed.

### Reporting summary

Further information on research design is available in the Nature Research Reporting Summary linked to this article.

## Supplementary information

Supplementary information

Description of additional supplementary files

Supplementary data 1

Supplementary data 2

Reporting Summary

## Data Availability

Source data files are provided in Supplementary Data 1 and 2.

## References

[1] V’kovski P, Kratzel A, Steiner S, Stalder H, Thiel V (2020). Coronavirus biology and replication: implications for SARS-CoV-2. Nat. Rev. Microbiol..

[2] Wu F (2020). A new coronavirus associated with human respiratory disease in China. Nature.

[3] Zhang L (2020). Crystal structure of SARS-CoV-2 main protease provides a basis for design of improved alpha-ketoamide inhibitors. Science.

[4] Mariano G, Farthing RJ, Lale-Farjat SLM, Bergeron JRC (2020). Structural characterization of SARS-CoV-2: where we are, and where we need to be. Front. Mol. Biosci..

[5] Wang C (2020). The establishment of reference sequence for SARS-CoV-2 and variation analysis. J. Med. Virol..

[6] Groneberg DA, Hilgenfeld R, Zabel P (2005). Molecular mechanisms of severe acute respiratory syndrome (SARS). Respir. Res..

[7] Chen S, Jonas F, Shen C, Hilgenfeld R (2010). Liberation of SARS-CoV main protease from the viral polyprotein: N-terminal autocleavage does not depend on the mature dimerization mode. Protein Cell.

[8] Ghahremanpour, M. M. et al. Identification of 14 known drugs as inhibitors of the main protease of SARS-CoV-2. *bioRxiv* (2020). 10.1021/acsmedchemlett.0c00521 PMC7605328 33324471

[9] Baker JD, Uhrich RL, Kraemer GC, Love JE, Kraemer BC (2021). A drug repurposing screen identifies hepatitis C antivirals as inhibitors of the SARS-CoV2 main protease. PLoS ONE.

[10] Kneller, D. W. et al. Structural, electronic, and electrostatic determinants for inhibitor binding to subsites S1 and S2 in SARS-CoV-2 main protease. *J. Med. Chem.* **64**, 17366–17383 (2021). 10.1021/acs.jmedchem.1c01475 PMC8565456 34705466

[11] Owen, D. R. et al. An oral SARS-CoV-2 M(pro) inhibitor clinical candidate for the treatment of COVID-19. *Science* **374**, eabl4784 (2021). 10.1126/science.abl4784 34726479

[12] Kneller DW (2022). Covalent narlaprevir- and boceprevir-derived hybrid inhibitors of SARS-CoV-2 main protease. Nat. Commun..

[13] Nashed NT, Aniana A, Ghirlando R, Chiliveri SC, Louis JM (2022). Modulation of the monomer-dimer equilibrium and catalytic activity of SARS-CoV-2 main protease by a transition-state analog inhibitor. Commun. Biol..

[14] Wang H (2020). Comprehensive insights into the catalytic mechanism of middle east respiratory syndrome 3C-like protease and severe acute respiratory syndrome 3C-like protease. ACS Catal..

[15] Ziebuhr J, Snijder EJ, Gorbalenya AE (2000). Virus-encoded proteinases and proteolytic processing in the Nidovirales. J. Gen. Virol..

[16] Allaire M, Chernaia MM, Malcolm BA, James MN (1994). Picornaviral 3C cysteine proteinases have a fold similar to chymotrypsin-like serine proteinases. Nature.

[17] Anand K (2002). Structure of coronavirus main proteinase reveals combination of a chymotrypsin fold with an extra alpha-helical domain. EMBO J..

[18] Fan K (2004). Biosynthesis, purification, and substrate specificity of severe acute respiratory syndrome coronavirus 3C-like proteinase. J. Biol. Chem..

[19] Muramatsu T (2013). Autoprocessing mechanism of severe acute respiratory syndrome coronavirus 3C-like protease (SARS-CoV 3CLpro) from its polyproteins. FEBS J..

[20] Muramatsu T (2016). SARS-CoV 3CL protease cleaves its C-terminal autoprocessing site by novel subsite cooperativity. Proc. Natl Acad. Sci. USA.

[21] Hsu MF (2005). Mechanism of the maturation process of SARS-CoV 3CL protease. J. Biol. Chem..

[22] Zhong N (2008). Without its N-finger, the main protease of severe acute respiratory syndrome coronavirus can form a novel dimer through its C-terminal domain. J. Virol..

[23] Shi J, Wei Z, Song J (2004). Dissection study on the severe acute respiratory syndrome 3C-like protease reveals the critical role of the extra domain in dimerization of the enzyme: defining the extra domain as a new target for design of highly specific protease inhibitors. J. Biol. Chem..

[24] Shi J, Song J (2006). The catalysis of the SARS 3C-like protease is under extensive regulation by its extra domain. FEBS J..

[25] Xia B, Kang X (2011). Activation and maturation of SARS-CoV main protease. Protein Cell.

[26] Goyal B, Goyal D (2020). Targeting the dimerization of the main protease of Coronaviruses: a potential broad-spectrum therapeutic strategy. ACS Comb. Sci..

[27] Shi J, Sivaraman J, Song J (2008). Mechanism for controlling the dimer-monomer switch and coupling dimerization to catalysis of the severe acute respiratory syndrome coronavirus 3C-like protease. J. Virol..

[28] Chen S (2008). Mutation of Gly-11 on the dimer interface results in the complete crystallographic dimer dissociation of severe acute respiratory syndrome coronavirus 3C-like protease: crystal structure with molecular dynamics simulations. J. Biol. Chem..

[29] Hu T (2009). Two adjacent mutations on the dimer interface of SARS coronavirus 3C-like protease cause different conformational changes in crystal structure. Virology.

[30] Cheng SC, Chang GG, Chou CY (2010). Mutation of Glu-166 blocks the substrate-induced dimerization of SARS coronavirus main protease. Biophys. J..

[31] Wu CG (2013). Mechanism for controlling the monomer-dimer conversion of SARS coronavirus main protease. Acta Crystallogr. D. Biol. Crystallogr..

[32] Chou CY (2004). Quaternary structure of the severe acute respiratory syndrome (SARS) coronavirus main protease. Biochemistry.

[33] Kneller DW (2020). Malleability of the SARS-CoV-2 3CL M(pro) active-site cavity facilitates binding of clinical antivirals. Structure.

[34] Noske GD (2021). A crystallographic snapshot of SARS-CoV-2 main protease maturation process. J. Mol. Biol..

[35] Grum-Tokars V, Ratia K, Begaye A, Baker SC, Mesecar AD (2008). Evaluating the 3C-like protease activity of SARS-Coronavirus: recommendations for standardized assays for drug discovery. Virus Res..

[36] Wang YC (2020). Structural basis of SARS-CoV-2 main protease inhibition by a broad-spectrum anti-coronaviral drug. Am. J. Cancer Res..

[37] Vuong W (2020). Feline coronavirus drug inhibits the main protease of SARS-CoV-2 and blocks virus replication. Nat. Commun..

[38] Chin J (1991). Developing artificial hydrolytic metalloenzymes by a unified mechanistic approach. J. Am. Chem. Soc..

[39] Kneller DW (2020). Unusual zwitterionic catalytic site of SARS-CoV-2 main protease revealed by neutron crystallography. J. Biol. Chem..

[40] Kneller DW, Zhang Q, Coates L, Louis JM, Kovalevsky A (2021). Michaelis-like complex of SARS-CoV-2 main protease visualized by room-temperature X-ray crystallography. IUCrJ.

[41] Chaturvedi RK, MacMahon AE, Schmir G (1967). Hydrolysis of thioimidate esters. Tetrahedral intermediates and general acid catalysis. J. Am. Chem. Soc..

[42] Campbell P, Nashed NT (1982). Carboxypeptidase A catalyzed hydrolysis of thiopeptide and thionester analogs of specific substrates. An effect on k_cat_ for peptide, but not ester, substrates. J. Am. Chem. Soc..

[43] Campbell P, Nashed NT, Lapinskas BA, Gurrieri J (1983). Thionesters as a probe for electrophilic catalysis in the serine protease mechanism. J. Biol. Chem..

[44] Moon JB, Coleman RS, Hanzlik RP (1986). Reversible covalent inhibition of papain by a peptide nitrile. Carbon-13 NMR evidence for a thioimidate ester adduct. J. Am. Chem. Soc..

[45] Gour-Salin BJ, Lachance P, Storer AC (1991). Inhibition of papain by peptide nitriles: conversion of the nitrile group into other functionalities via the papain:nitrile thioimidate ester adduct. Can. J. Chem..

[46] Loser R, Gutschow M (2009). Dipeptide-derived nitriles containing additional electrophilic sites: potentially irreversible inhibitors of cysteine proteases. J. Enzym. Inhib. Med. Chem..

[47] Silvestrini L (2021). The dimer-monomer equilibrium of SARS-CoV-2 main protease is affected by small molecule inhibitors. Sci. Rep..

[48] Lucast LJ, Batey RT, Doudna JA (2001). Large-scale purification of a stable form of recombinant tobacco etch virus protease. Biotechniques.

[49] Zhao, H., Brautigam, C. A., Ghirlando, R. & Schuck P. Overview of current methods in sedimentation velocity and sedimentation equilibrium analytical ultracentrifugation. *Curr. Protoc. Protein Sci.* Chapter 20:Unit20.12 (2013). 10.1002/0471140864.ps2012s71 PMC3652391 23377850

[50] Schuck P (2000). Size-distribution analysis of macromolecules by sedimentation velocity ultracentrifugation and Lamm equation modeling. Biophys. J..

[51] Cole JL, Lary JW, Moody TP, Laue TM (2008). Analytical ultracentrifugation: sedimentation velocity and sedimentation equilibrium. Methods Cell Biol..

[52] Schuck P (2003). On the analysis of protein self-association by sedimentation velocity analytical ultracentrifugation. Anal. Biochem..

[53] Brautigam CA (2015). Calculations and publication-quality illustrations for analytical ultracentrifugation data. Methods Enzymol..

[54] Liu YY (1999). Use of a fluorescence plate reader for measuring kinetic parameters with inner filter effect correction. Anal. Biochem..

[55] Evans PR, Murshudov GN (2013). How good are my data and what is the resolution?. Acta Crystallogr. D. Biol. Crystallogr..

[56] Winn MD (2011). Overview of the CCP4 suite and current developments. Acta Crystallogr. D. Biol. Crystallogr..

[57] McCoy AJ (2007). Phaser crystallographic software. J. Appl. Crystallogr..

[58] Liebschner D (2019). Macromolecular structure determination using X-rays, neutrons and electrons: recent developments in Phenix. Acta Crystallogr. D. Struct. Biol..

[59] Casanal A, Lohkamp B, Emsley P (2020). Current developments in Coot for macromolecular model building of electron cryo-microscopy and crystallographic data. Protein Sci..

[60] Chen VB (2010). MolProbity: all-atom structure validation for macromolecular crystallography. Acta Crystallogr. D. Biol. Crystallogr..

[61] Moriarty NW, Grosse-Kunstleve RW, Adams PD (2009). Electronic ligand builder and optimization workbench (eLBOW): a tool for ligand coordinate and restraint generation. Acta Crystallogr. D. Biol. Crystallogr..

[62] Frisch, M. J. et al. *Gaussian 16, Revision B.01* (Gaussian Inc., 2016).

[63] Yau WM, Tycko R (2018). Depletion of amyloid-beta peptides from solution by sequestration within fibril-seeded hydrogels. Protein Sci..

