# Supplementary material for: Autoprocessing and oxyanion loop reorganization upon GC373 and nirmatrelvir binding of monomeric SARS-CoV-2 main protease catalytic domain
Source: Commun Biol. 2022 Sep 16;5:976. doi: 10.1038/s42003-022-03910-y (PMC9481597; doi:10.1038/s42003-022-03910-y)
Supplement: Supplementary file 1 — Supplementary information [file 42003_2022_3910_MOESM1_ESM.docx]

Supplementary information

**Autoprocessing and oxyanion loop reorganization upon GC373 and nirmatrelvir binding of monomeric SARS-CoV-2 main protease catalytic domain**

Nashaat T. Nashed,^1^ Daniel W. Kneller,^2^ Leighton Coates,^3^ Rodolfo Ghirlando,^4^ Annie Aniana,^1^ Andrey Kovalevsky,^2*^ John M. Louis^1*^

*^1^Laboratory of Chemical Physics, National Institute of Diabetes and Digestive and Kidney Diseases, National Institutes of Health, DHHS****,*** *Bethesda, MD 20892-0520, USA*

*^2^Neutron Scattering Division, Oak Ridge National Laboratory, 1 Bethel Valley Road, Oak Ridge, TN, 37831, USA*

*^3^Second Target Station, Oak Ridge National Laboratory, 1 Bethel Valley Road, Oak Ridge, TN, 37831, USA*

*^4^Laboratory of Molecular Biology, National Institute of Diabetes and Digestive and Kidney Diseases, National Institutes of Health, DHHS****,*** *Bethesda, MD 20892-0520, USA*

* Correspondence to Andrey Kovalevsky ([kovalevskyay@ornl.gov](mailto:kovalevskyay@ornl.gov)) and John M. Louis ([johnl@niddk.nih.gov](mailto:johnl@niddk.nih.gov))

**Table S1. Estimated k_cat_/K_m_, ΔG, K_dimer_.app and K_d_ by enzyme kinetics, SV-AUC and ITC of MPro^WT^ and its analogues.**

| **Chemical structure** | **Construct** | **k_cat_/K_m_, (µM^-1^ min^-1^)** | **-ΔG (kcal/mol)** | **K_dimer_.app (µM)** | **K_d_ = K_i_  (µM)** | **Difference K_dimer_/K_d_** |
| --- | --- | --- | --- | --- | --- | --- |
| no inhibitor | MPro^WT^ | 0.6±0.05 | 0.306 | 2^(c)^ |  |  |
|  | MPro^M^ | (5±0.6)10^-3^ | 3.2 | >90 |  |  |
|  | MPro^1-199^ | (1.3±0.02)10^-6(a)^  (1.0±0.03)10^-6(b)^ | 8.0  8.1 | >1200 |  |  |
| GC373   | MPro^WT^ |  |  | ≤2^(d)^ | 0.15 ± 0.03^(e)^ |  |
|  | MPro^C145A^ |  |  |  | no response |  |
|  | MPro^M^ |  |  | 6.2^(e)^ | 6.2^(e)^ | 1 |
|  | MPro^1-199^ |  |  | 1200 ± 200 | 32 ± 5 | 38 |
|  | MPro^1-196^ |  |  | >1200 | 45 ± 20 | 27 |
|  | MPro^10-306^ |  |  | >>1200 | 44 ± 8 | 27 |
| NMV   | MPro^WT^ |  |  | ≤2^(d)^ | 0.007 ± 0.003^(f)^ | <(3.5)10^-3^ |
|  | MPro^C145A^ |  |  |  | 2.7 ± 0.9 |  |
|  | MPro^1-199^ |  |  | 180 ± 0.01 | 19 ± 3 | 9 |
|  | MPro^1-199(C145A)^ |  |  | >200 | no response |  |
|  | MPro^1-196^ |  |  | 510 ± 0.05 | 14 ± 3 | 36 |
|  | MPro^10-306^ |  |  | >510 | no response |  |

Enzyme kinetics were carried out as described previously in reference 1. ^(a)^Calculated from Lineweaver-Burk plot. ^(b)^Calculated from plot of v *vs* [E]. Activities of MPro^WT^ and MPro^M^ are cited from reference.^1^ ΔG was calculated according to the equation ΔG = -RTlnK. Approximate K_dimer_.app and K_d_ were derived from SV-AUC and ITC analyses, respectively. ^(c)^Cited from reference.^2^ ^(d)^The K_dimer_ for MPro^WT^ in the presence of inhibitor is predicted to be 2 µM or lower because of the thermodynamic stabilization of the dimer upon inhibitor binding. ^(e)(f)^Cited from references ^1^ and ^3^, respectively, solely for comparison. For competitive inhibitors that bind at only one site, the dissociation constant (K_d_ = 1/K_a_) is equivalent to the inhibition constant measured by enzyme kinetics (K_i_).

**Fig. S1.**

**Amino acid sequence of MPro constructs**

**MPro^WT^**

-6

~~MBP+ΔGB1+TSAVLQ~~

10 20 30 40 50 60

SGFRKMAFPS GKVEGCMVQV TCGTTTLNGL WLDDVVYCPR HVICTSEDML NPNYEDLLIR

70 80 90 100 110 120

KSNHNFLVQA GNVQLRVIGH SMQNCVLKLK VDTANPKTPK YKFVRIQPGQ TFSVLACYNG

130 140 150 160 170 180

SPSGVYQCAM RPNFTIKGSF LNGSCGSVGF NIDYDCVSFC YMHHMELPTG VHAGTDLEGN

190 200 210 220 230 240

FYGPFVDRQT AQAAGTDTTI TVNVLAWLYA AVINGDRWFL NRFTTTLNDF NLVAMKYNYE

250 260 270 280 290 300

PLTQDHVDIL GPLSAQTGIA VLDMCASLKE LLQNGMNGRT ILGSALLEDE FTPFDVVRQC

306

SGVTFQ~~GPHHHHHH~~

**Calculated molecular weight:** 33796.8

**control MPro^C145A^**

10 20 30 40 50 60

SGFRKMAFPS GKVEGCMVQV TCGTTTLNGL WLDDVVYCPR HVICTSEDML NPNYEDLLIR

70 80 90 100 110 120

KSNHNFLVQA GNVQLRVIGH SMQNCVLKLK VDTANPKTPK YKFVRIQPGQ TFSVLACYNG

130 140 150 160 170 180

SPSGVYQCAM RPNFTIKGSF LNGSAGSVGF NIDYDCVSFC YMHHMELPTG VHAGTDLEGN

190 200 210 220 230 240

FYGPFVDRQT AQAAGTDTTI TVNVLAWLYA AVINGDRWFL NRFTTTLNDF NLVAMKYNYE

250 260 270 280 290 300

PLTQDHVDIL GPLSAQTGIA VLDMCASLKE LLQNGMNGRT ILGSALLEDE FTPFDVVRQC

306

SGVTFQ~~GPHHHHHH~~

**Calculated molecular weight:** 33764.7

**MPro^1-199^**

10 20 30 40 50 60

SGFRKMAFPS GKVEGCMVQV TCGTTTLNGL WLDDVVYCPR HVICTSEDML NPNYEDLLIR

70 80 90 100 110 120

KSNHNFLVQA GNVQLRVIGH SMQNCVLKLK VDTANPKTPK YKFVRIQPGQ TFSVLACYNG

130 140 150 160 170 180

SPSGVYQCAM RPNFTIKGSF LNGSCGSVGF NIDYDCVSFC YMHHMELPTG VHAGTDLEGN

190 199

FYGPFVDRQT AQAAGTDTTLEHHHHHH

**Calculated molecular weight:** 22950.2

**Control MPro^1-199(C145A)^**

10 20 30 40 50 60

SGFRKMAFPS GKVEGCMVQV TCGTTTLNGL WLDDVVYCPR HVICTSEDML NPNYEDLLIR

70 80 90 100 110 120

KSNHNFLVQA GNVQLRVIGH SMQNCVLKLK VDTANPKTPK YKFVRIQPGQ TFSVLACYNG

130 140 150 160 170 180

SPSGVYQCAM RPNFTIKGSF LNGSAGSVGF NIDYDCVSFC YMHHMELPTG VHAGTDLEGN

190 199

FYGPFVDRQT AQAAGTDTTLEHHHHHH

**Calculated molecular weight:** 22918.1

**MPro^1-196^** 11 21 31 41

~~GSSHHHHHHS SGENLYFQ~~GS GFRKMAFPSG KVEGCMVQVT CGTTTLNGLW LDDVVYCPRH

51 61 71 81 91 101

VICTSEDMLN PNYEDLLIRK SNHNFLVQAG NVQLRVIGHS MQNCVLKLKV DTANPKTPKY

111 121 131 141 151 161

KFVRIQPGQT FSVLACYNGS PSGVYQCAMR PNFTIKGSFL NGSCGSVGFN IDYDCVSFCY

171 181 191

MHHMELPTGV HAGTDLEGNF YGPFVDRQTA QAAGT

**Calculated molecular weight:** 21624.8

**MPro^10-306^**

10 20 30 40 50 60

S GKVEGCMVQV TCGTTTLNGL WLDDVVYCPR HVICTSEDML NPNYEDLLIR

70 80 90 100 110 120

KSNHNFLVQA GNVQLRVIGH SMQNCVLKLK VDTANPKTPK YKFVRIQPGQ TFSVLACYNG

130 140 150 160 170 180

SPSGVYQCAM RPNFTIKGSF LNGSCGSVGF NIDYDCVSFC YMHHMELPTG VHAGTDLEGN

190 200 210 220 230 240

FYGPFVDRQT AQAAGTDTTI TVNVLAWLYA AVINGDRWFL NRFTTTLNDF NLVAMKYNYE

250 260 270 280 290 300

PLTQDHVDIL GPLSAQTGIA VLDMCASLKE LLQNGMNGRT ILGSALLEDE FTPFDVVRQC

306

SGVTFQ~~GPHH HHHH~~

**Calculated molecular weight:** 32774.5

**^(-25)^MPro^1-199^**

-25 1 15

GSSHHHHHHS SGENLYFQGS NDFSNSGSDV LYQPPQTSIT SAVLQSGFRK MAFPSGKVEG

25 35 45 55 65 75

CMVQVTCGTT TLNGLWLDDV VYCPRHVICT SEDMLNPNYE DLLIRKSNHN FLVQAGNVQL

85 95 105 115 125 135

RVIGHSMQNC VLKLKVDTAN PKTPKYKFVR IQPGQTFSVL ACYNGSPSGV YQCAMRPNFT

145 155 165 175 185 195

IKGSFLNGSC GSVGFNIDYD CVSFCYMHHM ELPTGVHAGT DLEGNFYGPF VDRQTAQAAG

199

TDTTLEHHHH HH

**Calculated molecular weight:** 27825.3

**^(-25)^MPro^1-199(C145A)^ control**

-25 1 13 23 33

GSNDFSNSGS DVLYQPPQTS ITSAVLQSGF RKMAFPSGKV EGCMVQVTCG TTTLNGLWLD

43 53 63 73 83 93

DVVYCPRHVI CTSEDMLNPN YEDLLIRKSN HNFLVQAGNV QLRVIGHSMQ NCVLKLKVDT

103 113 123 133 143 153

ANPKTPKYKF VRIQPGQTFS VLACYNGSPS GVYQCAMRPN FTIKGSFLNG SAGSVGFNID

163 173 183 193 199

YDCVSFCYMH HMELPTGVHA GTDLEGNFYG PFVDRQTAQA AGTDTTLEHH HHHH

**Calculated molecular weight:** 25713.1

**^(-25)^MPro^1-196^**

-25 1 15

GSSHHHHHHS SGENLYFQGS NDFSNSGSDV LYQPPQTSIT SAVLQSGFRK MAFPSGKVEG

25 35 45 55 65 75

CMVQVTCGTT TLNGLWLDDV VYCPRHVICT SEDMLNPNYE DLLIRKSNHN FLVQAGNVQL

85 95 105 115 125 135

RVIGHSMQNC VLKLKVDTAN PKTPKYKFVR IQPGQTFSVL ACYNGSPSGV YQCAMRPNFT

155 165 175 185 195

IKGSFLNGSC GSVGFNIDYD CVSFCYMHHM ELPTGVHAGT DLEGNFYGPF VDRQTAQAAG T

**Calculated molecular weight:** 26442.9

**^(-25)^MPro^1-196(C145A)^ control**

-25 1 15

GSSHHHHHHS SGENLYFQGS NDFSNSGSDV LYQPPQTSIT SAVLQSGFRK MAFPSGKVEG

25 35 45 55 65 75

CMVQVTCGTT TLNGLWLDDV VYCPRHVICT SEDMLNPNYE DLLIRKSNHN FLVQAGNVQL

85 95 105 115 125 135

RVIGHSMQNC VLKLKVDTAN PKTPKYKFVR IQPGQTFSVL ACYNGSPSGV YQCAMRPNFT

155 165 175 185 195

IKGSFLNGSA GSVGFNIDYD CVSFCYMHHM ELPTGVHAGT DLEGNFYGPF VDRQTAQAAG T

**Calculated molecular weight:** 26410.8

**^(-25)^MPro^1-187^**

-25 1 5 15

GSSHHHHHHS SGENLYFQGS NDFSNSGSDV LYQPPQTSIT SAVLQSGFRK MAFPSGKVEG

25 35 45 55 65 75

CMVQVTCGTT TLNGLWLDDV VYCPRHVICT SEDMLNPNYE DLLIRKSNHN FLVQAGNVQL

85 95 105 115 125 135

RVIGHSMQNC VLKLKVDTAN PKTPKYKFVR IQPGQTFSVL ACYNGSPSGV YQCAMRPNFT

145 155 165 175 187

IKGSFLNGSC GSVGFNIDYD CVSFCYMHHM ELPTGVHAGT DLEGNFYGPF VDLEHHHH HH

**Calculated molecular weight:** 26623.1

**Fig. S1. Amino acid sequence of recombinant MPro constructs and their designations used in this study.** Non-native residues flanking the sequences and those removed via autoprocessing or during purification with TEV or HRV-3C proteases are underlined and struck through, respectively. Active site C145A mutation is shown in red. Theoretical mass of the purified protein is indicated below each sequence of the corresponding construct. ^(-25)^ and ^(-6)^ denote 6 and 25 amino acids of the C-terminal residues of nsp4 flanking the N-terminus of nsp5 (MPro) appended to MPro and its analogues.

**Fig. S2.**


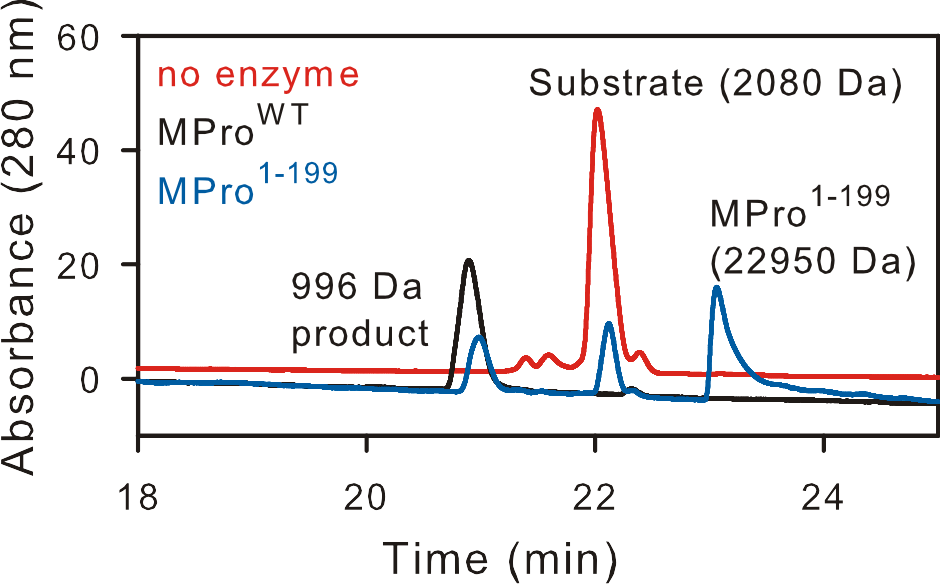


**Fig. S2. RPLC-MS analyses of the product upon cleavage of the FRET substrate catalyzed by MPro^WT^ (black) and MPro^1-196^ (blue).** Following the digest with MPro^WT^ and MPro^1-199^ of the FRET substrate, the sample was diluted in 5% acetic acid and subjected to RPLC-MS and data processed as described in Methods. Only one of the products elutes from the column under these conditions.

**Fig. S3.**


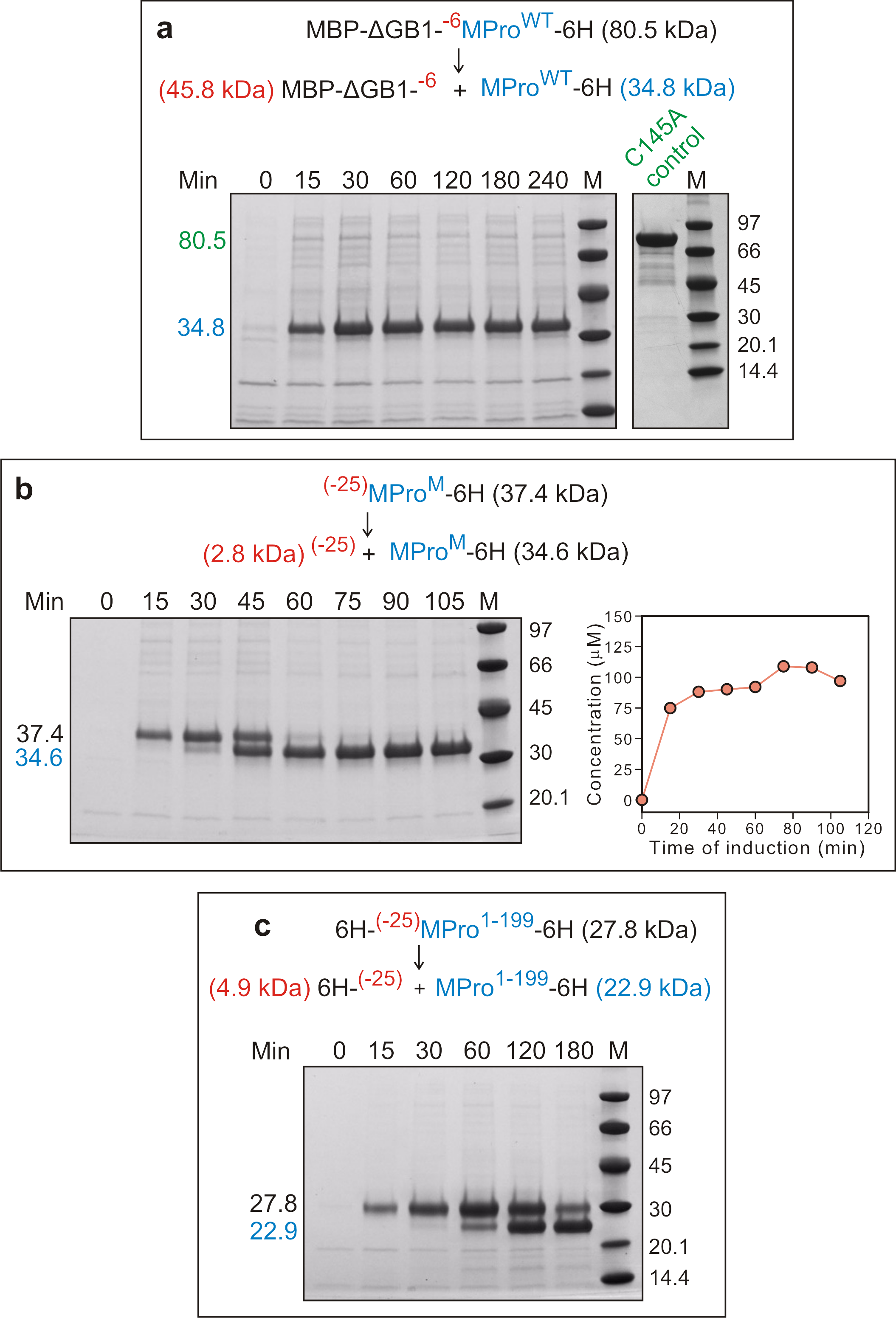


**Fig. S3. N-terminal autoprocessing of various MPro^WT^, MPro^M^ and MPro^1-199^ precursor constructs.** A schematic of the construct consisting of 6 or 25 amino acids of the flanking C-terminal residues of nsp4 appended to the N-terminus of (a) MPro^WT^, (b) MPro^M^ and (c) MPro^1-199^ is shown above each gel panel. The N-terminal cleavage site is indicated with a downward black arrow. The precursor, products released upon cleavage at the N-terminus of MPro and molecular weight standards (M) are indicated in kDa. Following addition of IPTG for protein expression, 12 ml of cells were harvested at the time points indicated above the lanes, subjected to NAC and equal volume of the bound fractions were analyzed by SDS-PAGE, as described in Experimental Section. Autoprocessing for the wild-type precursor (a) is fastest, resulting in products (34.8 kDa) within 15 min of induction with no indication of the presence of the precursor (80.5 kDa), whereas MPro^M^ ^1^ (b) is slower relative to wild-type and MPro^1-199^ (c) being the slowest. MBP and ΔGB1 denote the maltose binding protein and a truncated immunoglobulin binding domain B1 of protein G, respectively.^1, 4^ A control construct bearing the C145A mutation is shown in green (a). A plot of the protein concentration as a function of time of induction is also shown in (b). The concentration of the accumulated protein was estimated from the optical density which was then converted to volume for a constant volume of culture from which the proteins were purified.^5^ Accumulation reaches a maximum of ~100 µM within 20 min of induction for MPro^M^.

**Fig. S4.**


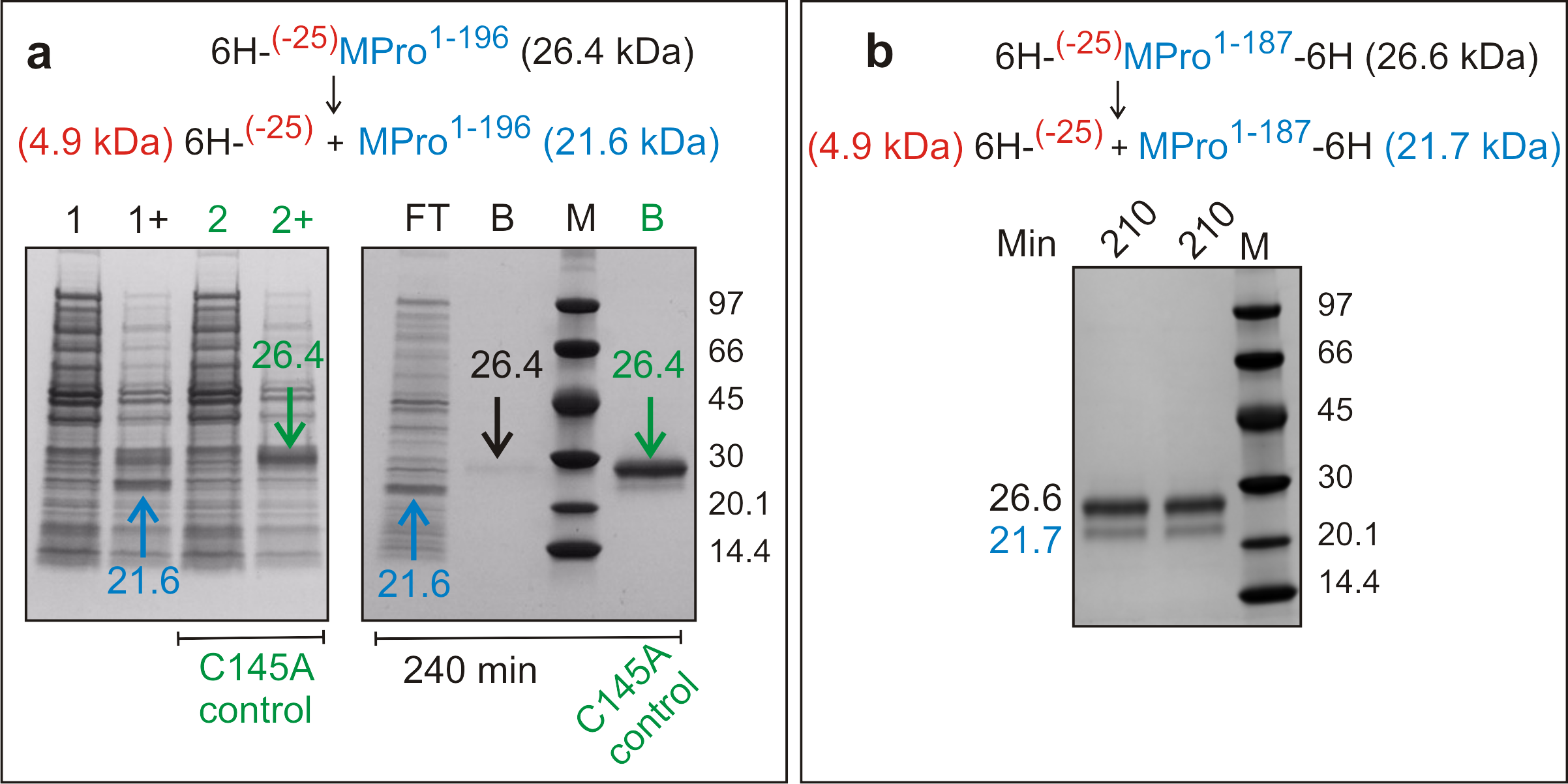


**Fig. S4. N-terminal autoprocessing of MPro^1-196^ and MPro^1-187^ precursor constructs.** A schematic of the construct consisting of 25 amino acids of the flanking C-terminal residues of nsp4 appended to the N-terminus of (a) MPro^1-196^ and (b) MPro^1-187^ is shown above each gel panel. The N-terminal cleavage site is indicated with a downward black arrow. The precursor, products released upon cleavage at the N-terminus of MPro and molecular weight standards (M) are indicated in kDa. (a) As this construct does not carry a 6His-tag at the C-terminus, to mimic that of the mature counterpart MPro^1-196^, the processed MPro^1-196^ resulting from cleavage at the nsp4/nsp5 junction is not retained after NAC and thus, is observed in the flow through (FT, black). Very little of the unprocessed miniprecursor is present in the bound (B, black) fraction. A control of the same construct bearing a C145A mutation to preclude autoprocessing is observed in the bound (B, green) fraction as expected. Total extracts of these constructs before and after (+) 240 min induction are shown on the left panel. Lanes 1 and 2 correspond to the construct undergoing autoprocessing (black) compared to the active site mutant C145A (green). (b) The same construct design as in Fig. S3B and S3C with a region spanning residues 1-187 of MPro shows restricted processing because it lacks the S5 loop residues 188-194.

**Fig. S5.**


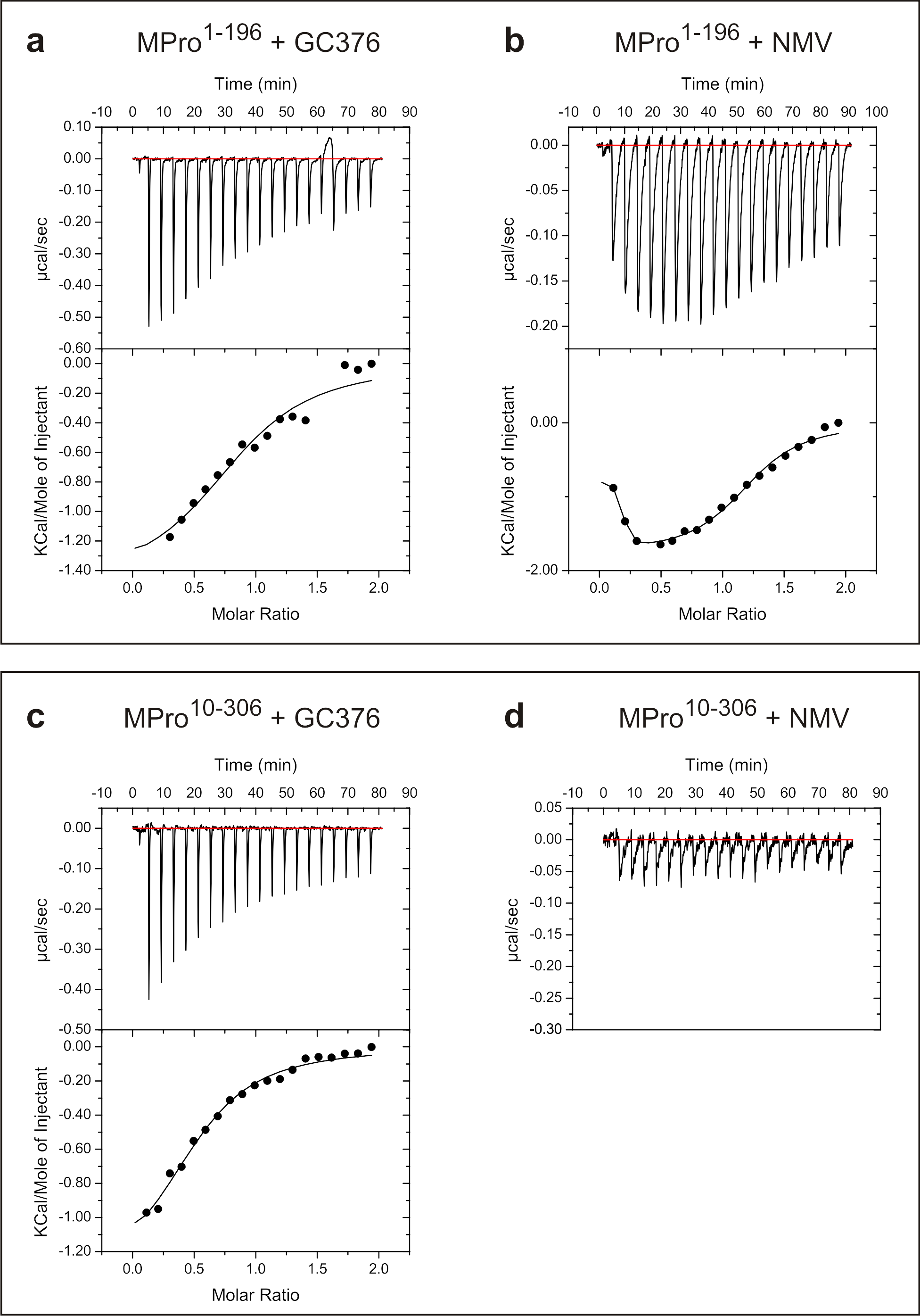


**Fig. S5. Binding isotherms of GC373 and NMV to MPro^1-196^ and MPro^10-306^.** Titrations were carried out in buffer C at 28 °C. The thermodynamic parameters are listed in Table 1.

**Fig. S6.**


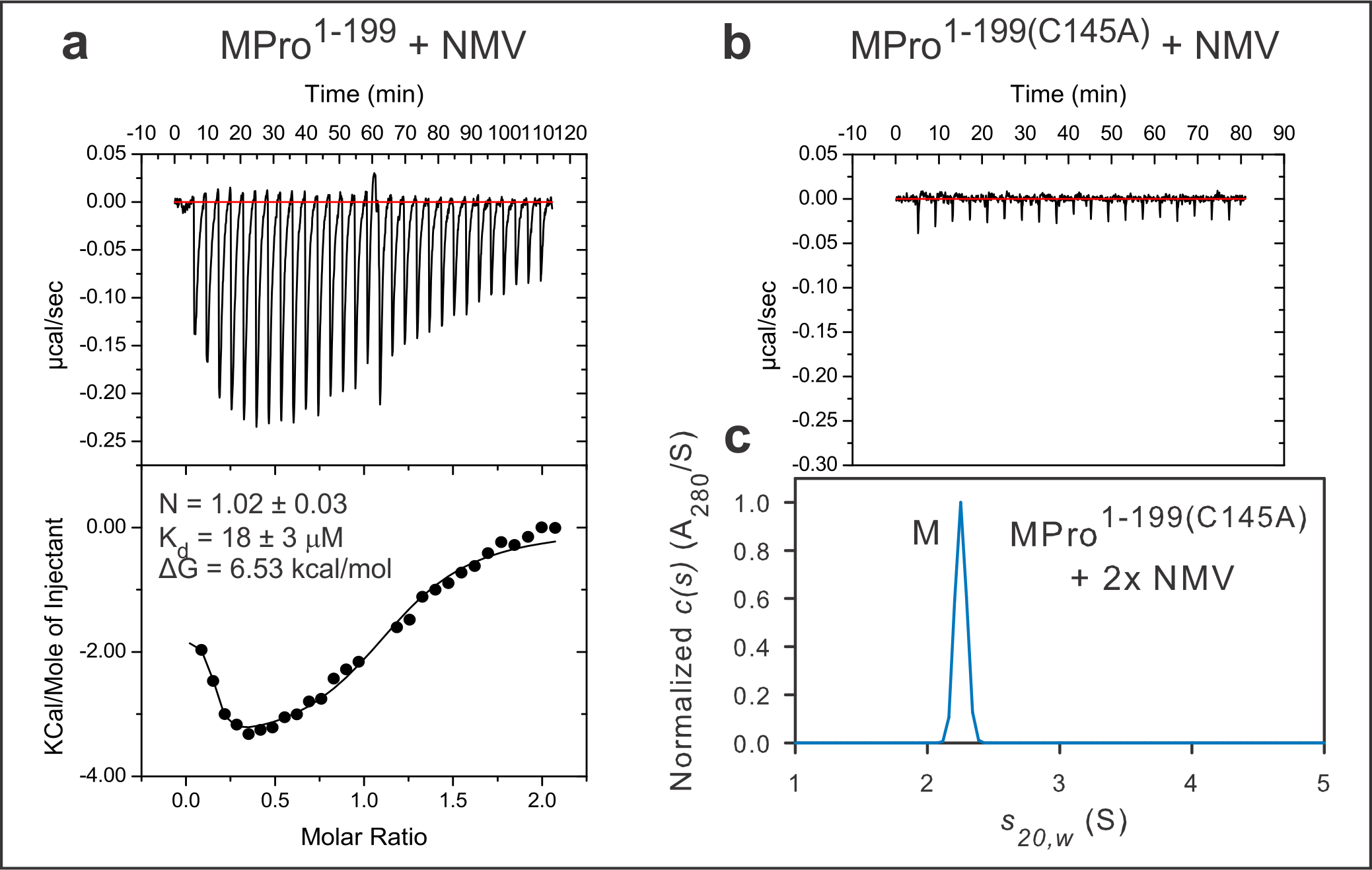


**Fig. S6. Binding isotherms of NMV to MPro^1-196^ and MPro^10-306^.** Titrations were carried out in buffer C at 28 °C. (a) MPro^1-199^ *vs* NMV is a duplicate titration with 30 injections instead of the 20 injections shown in Fig. 3C validating the two processes of binding followed by slow reactivity which dominates the isotherm. (b) No thermal response was observed when titrating NMV (3 mM in syringe) with MPro^1-199(C145A)^ (300 µM in cell) suggestive of very weak or no binding under the conditions employed. This result is consitent with RPLC-MS data showing the absence of the imidate thioester adduct of NMV (Fig. 7b) as compared to MPro^1-199^ in the presence of NMV (Fig. 7c and d) as well as dimer formation detectable only upon the binding of NMV to MPro^1-199^ by SV-AUC [compare Fig. 6b (red trace) with S6c above (blue trace)]. (c) SV-AUC analysis of ~250 µM MPro^1-199(C145A)^ in the presence of 2x NMV recovered after ITC.

**Fig. S7.**


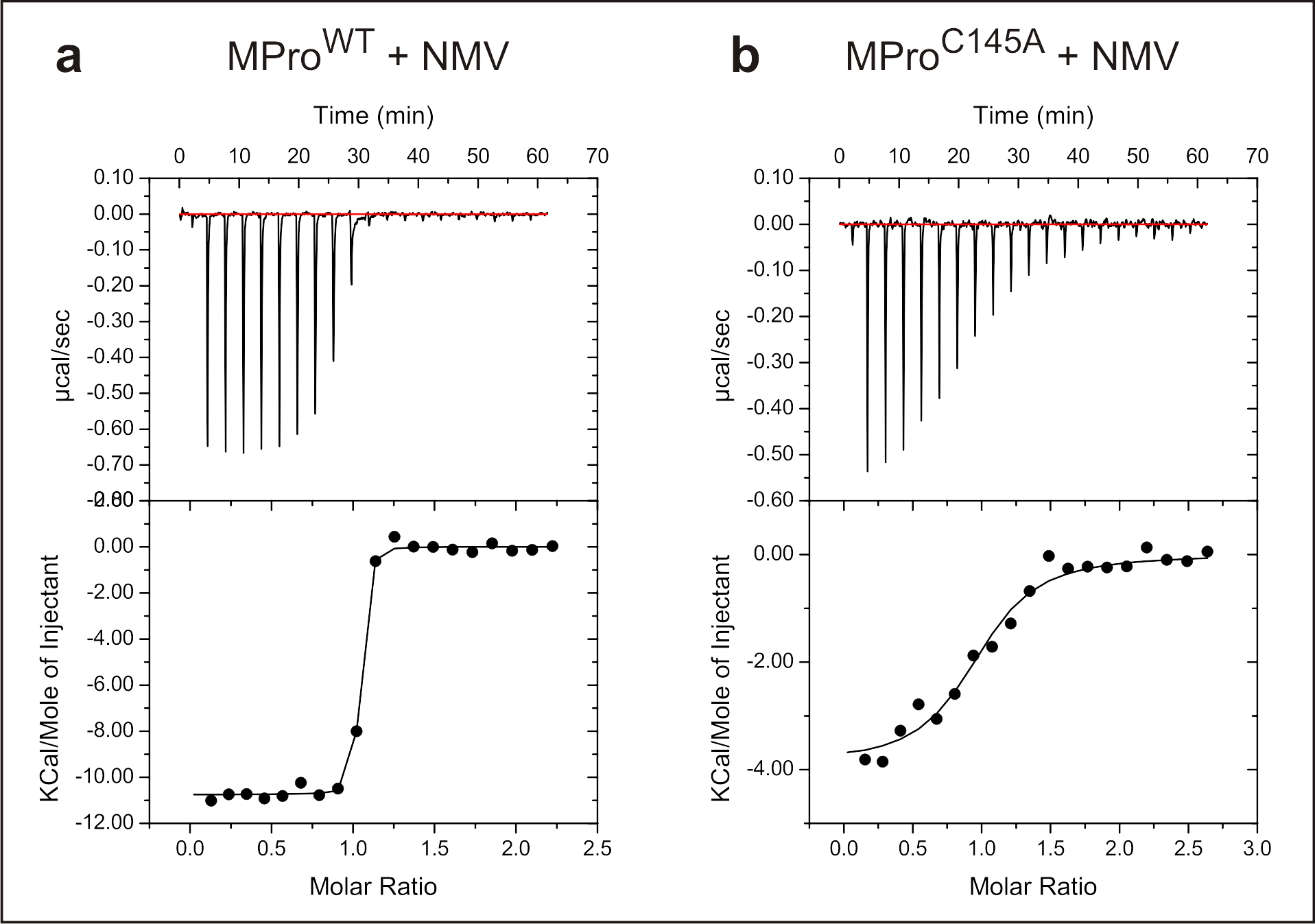


**Fig. S7. Binding isotherms of NMV to MPro^WT^ and MPro^C145A^.** Titrations were carried out in buffer C at 28 °C. The thermodynamic parameters are listed in Table 1. Our MPro^WT^ data is reproduced here from reference^3^ for ease of comparison with MPro^C145A^ (this work).

**Fig. S8.**


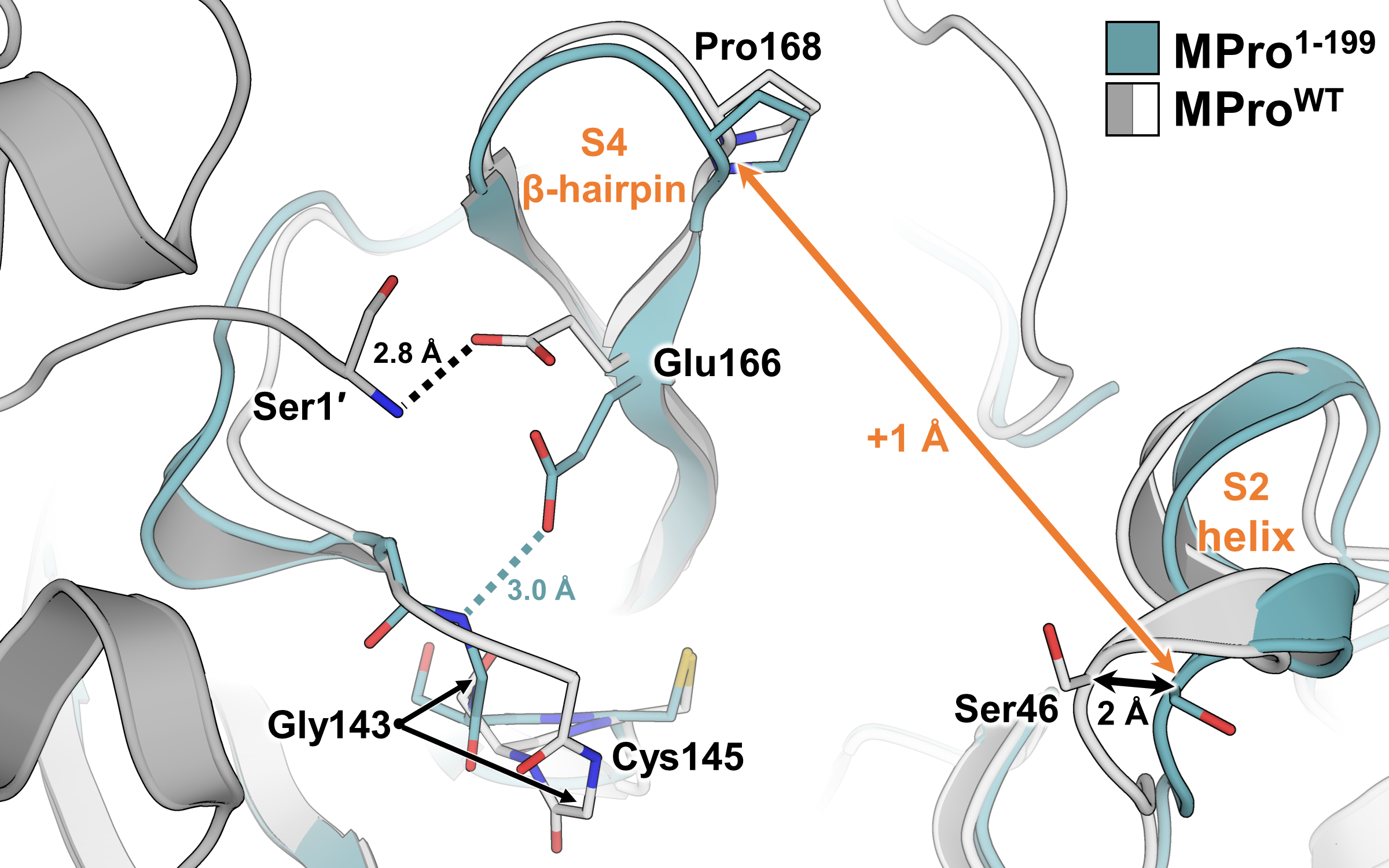


**Fig. S8. Superposition of MPro^1-199^ with MPro^WT^.** Structural organization of MPro^1-199^ active site relative to MPro^WT^ dimer (PDB ID 7JUN) displays conformational differences. In MPro^1-199^ relative to MPro^WT^, Glu166 is rotated to H-bond with Gly143 of the unwound oxyanion loop. The span between the S4 β-hairpin and S2 helix is ~1 Å longer in MPro^1-199^ than in the mature dimer.

**Fig. S9.**


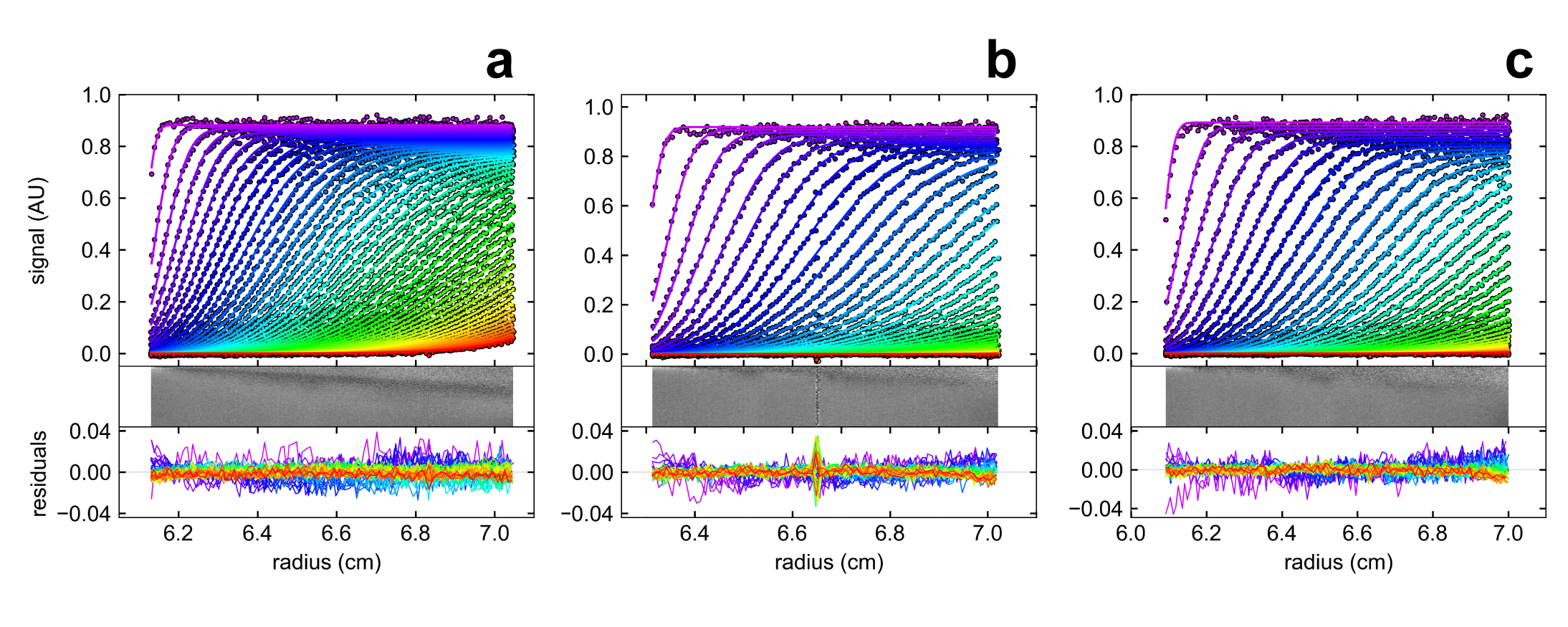


**Fig. S9. Estimation of the apparent K_dimer_ (K_d_.app) of MPro^1-199^ and MPro^1-196^ in the presence of inhibitors by Lamm equation modeling.** Sedimentation velocity absorbance data collected at 50,000 rpm and 25°C in 3 mm pathlength cells for (a) 191 µM MPro^1-199^ with GC373 inhibitor with 150 scans collected over 7.5 hours, (b) 195 µM MPro^1-199^ with NMV inhibitor with 140 scans collected over 8.7 hours, and (c) 186 µM MPro^1-196^ in the presence of NMV inhibitor with 120 scans collected over 9.8 hours. Together with the interference scans, data were analyzed in terms of a reversible monomer-dimer self-association model. For clarity only every third scan and every third experimental data point are shown. Best-fits are represented by a solid line through the experimental points. A bitmap representation of the residuals to the best-fit, together with the combined residuals, are shown below each plot. Based on the loading concentrations and dissociation constants the following dimer contributions (in monomer units) were determined for each of the constructs. MPro^1-199^ with GC373 has a 20% dimer contribution. MPro^1-199^ with NMV has 52% dimer, and MPro^1-196^ in the presence of NMV has 33% dimer.

**Fig. S10.**


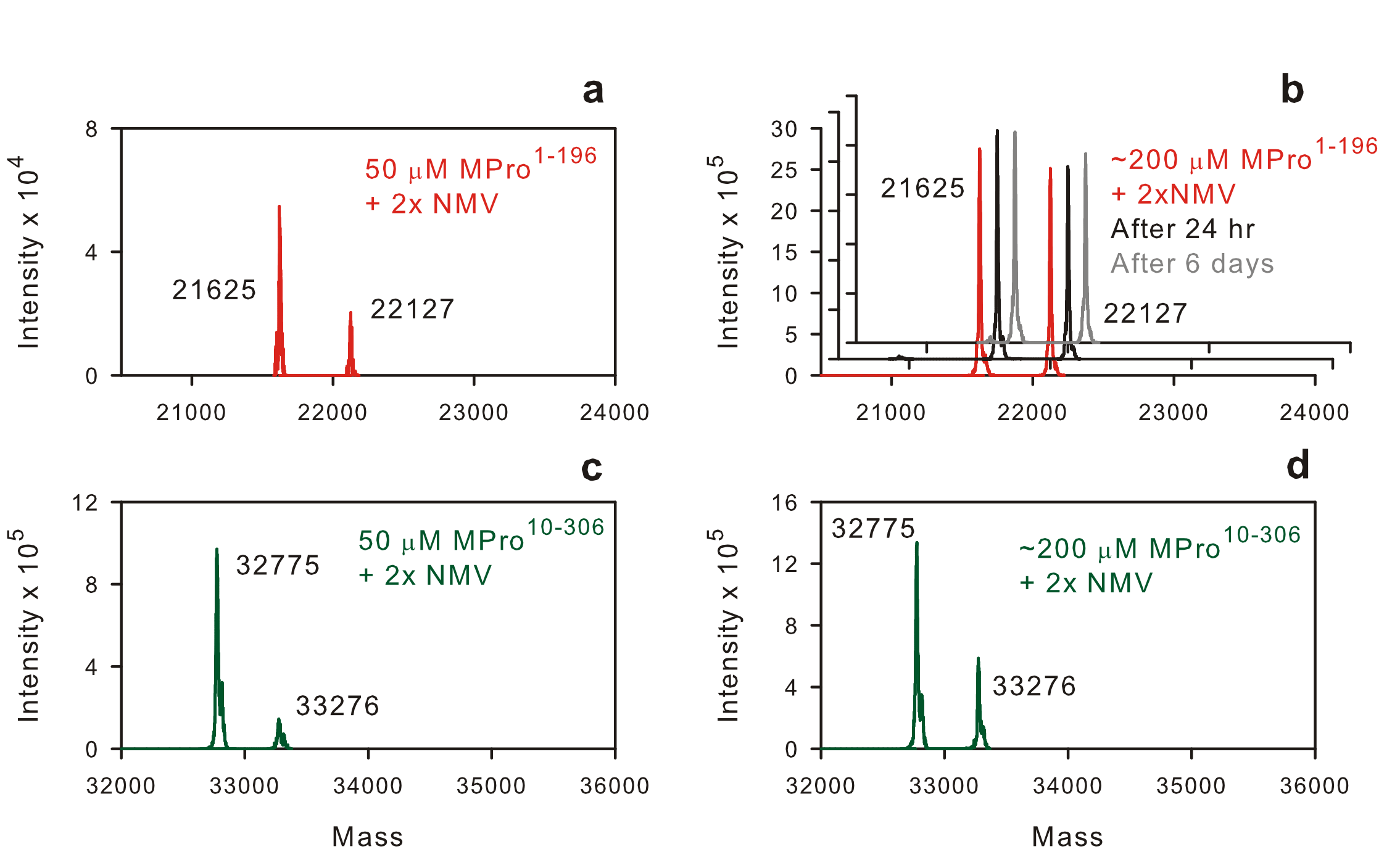


**Fig. S10. Mass spectra of NMV adduct bound to MPro^1-196^ and MPro^10-306^.** (a, c) Proteins were mixed with NMV at the indicated concentrations. (b, d) Samples recovered from the cell after ITC. Actual concentration of b and d are as shown for SV-AUC plots in Fig. 6d and e. All samples were diluted to a concentration of 20 µM in 5% acetic acid and 10 µl of each was subjected to RPLC fractionation coupled to a mass spectrometry detector. Data were processed with software provided with the instrument and plots were generated with Sigmaplot. Calculated mass of the construct used is shown in Fig. S1 under each amino acid sequence of the corresponding construct. Black and gray traces in panel B indicate samples upon dilution to 20 µM and incubation for 24 hr and 6 days, respectively, prior to subjecting 10 µl to RPLC-MS.

References

1. Nashed NT, Aniana A, Ghirlando R, Chiliveri SC, Louis JM. Modulation of the monomer-dimer equilibrium and catalytic activity of SARS-CoV-2 main protease by a transition-state analog inhibitor. *Commun Biol* **5**, 160 (2022).

2. Zhang L*, et al.* Crystal structure of SARS-CoV-2 main protease provides a basis for design of improved alpha-ketoamide inhibitors. *Science* **368**, 409-412 (2020).

3. Kneller DW*, et al.* Covalent narlaprevir- and boceprevir-derived hybrid inhibitors of SARS-CoV-2 main protease. *Nat Commun* **13**, 2268 (2022).

4. Kneller DW*, et al.* Structural, Electronic, and Electrostatic Determinants for Inhibitor Binding to Subsites S1 and S2 in SARS-CoV-2 Main Protease. *J Med Chem*, (2021).

5. Volkmer B, Heinemann M. Condition-dependent cell volume and concentration of Escherichia coli to facilitate data conversion for systems biology modeling. *PLoS One* **6**, e23126 (2011).
