# Supplementary material for: Autoprocessing and oxyanion loop reorganization upon GC373 and nirmatrelvir binding of monomeric SARS-CoV-2 main protease catalytic domain
Source: Commun Biol. 2022 Sep 16;5:976. doi: 10.1038/s42003-022-03910-y (PMC9481597; doi:10.1038/s42003-022-03910-y)
Supplement: Supplementary file 2 — Description of additional supplementary files [file 42003_2022_3910_MOESM2_ESM.docx]

**Description of additional supplementary files**

**File name:** Supplementary data 1

**Description:** Source data for all main figures

**File name:** Supplementary data 2

**Description:** Source data for all supplementary information figures
