# Supplementary material for: Autoprocessing and oxyanion loop reorganization upon GC373 and nirmatrelvir binding of monomeric SARS-CoV-2 main protease catalytic domain
Source: Commun Biol. 2022 Sep 16;5:976. doi: 10.1038/s42003-022-03910-y (PMC9481597; doi:10.1038/s42003-022-03910-y)
Supplement: Supplementary file 5 — Reporting Summary [file 42003_2022_3910_MOESM5_ESM.pdf]

nature portfolio

Corresponding author(s): COMMSBIO-22-2101-A

Last updated by author(s): Aug 22, 2022

Reporting Summary

Nature Portfolio wishes to improve the reproducibility of the work that we publish. This form provides structure for consistency and transparency in reporting. For further information on Nature Portfolio policies, see our Editorial Policies and the Editorial Policy Checklist.

Statistics

For all statistical analyses, confirm that the following items are present in the figure legend, table legend, main text, or Methods section.

n/a	Confirmed	
☒	☐	The exact sample size (*n*) for each experimental group/condition, given as a discrete number and unit of measurement
☒	☐	A statement on whether measurements were taken from distinct samples or whether the same sample was measured repeatedly
☒	☐	The statistical test(s) used AND whether they are one- or two-sided<br>*Only common tests should be described solely by name; describe more complex techniques in the Methods section.*
☒	☐	A description of all covariates tested
☒	☐	A description of any assumptions or corrections, such as tests of normality and adjustment for multiple comparisons
☒	☐	A full description of the statistical parameters including central tendency (e.g. means) or other basic estimates (e.g. regression coefficient) AND variation (e.g. standard deviation) or associated estimates of uncertainty (e.g. confidence intervals)
☒	☐	For null hypothesis testing, the test statistic (e.g. *F*, *t*, *r*) with confidence intervals, effect sizes, degrees of freedom and *P* value noted<br>*Give P values as exact values whenever suitable.*
☒	☐	For Bayesian analysis, information on the choice of priors and Markov chain Monte Carlo settings
☒	☐	For hierarchical and complex designs, identification of the appropriate level for tests and full reporting of outcomes
☒	☐	Estimates of effect sizes (e.g. Cohen's *d*, Pearson's *r*), indicating how they were calculated

Our web collection on statistics for biologists contains articles on many of the points above.

Software and code

Policy information about availability of computer code

Data collection	CrysAlis Pro 41_64.113a
Data analysis	CCP4 7.0.021, Phenix 1.18, Coot 0.8.2, GraphPad 9.3.1, Origin2019, Sigmaplot 10, Astra 7

For manuscripts utilizing custom algorithms or software that are central to the research but not yet described in published literature, software must be made available to editors and reviewers. We strongly encourage code deposition in a community repository (e.g. GitHub). See the Nature Portfolio guidelines for submitting code & software for further information.

Data

Policy information about availability of data

All manuscripts must include a data availability statement. This statement should provide the following information, where applicable:
- Accession codes, unique identifiers, or web links for publicly available datasets
- A description of any restrictions on data availability
- For clinical datasets or third party data, please ensure that the statement adheres to our policy

All data generated or analysed during this study are included in this published article (and its supplementary information files). The structure and corresponding structure factors have been deposited into the protein data bank with the PDB accession codes 7UJ9 for MPro1-199 [http://doi.org/10.2210/pdb7UJ9/pdb], 7UJG for MPro1-196-GC373 [http://doi.org/10.2210/pdb7UJG/pdb], 7UJU for MPro1-196-NMV [http://doi.org/10.2210/pdb7UJU/pdb], and 7UKK for MproWT-GC373 [http://doi.org/10.2210/pdb7UKK/pdb]. Supporting information is available online.

Field-specific reporting

Please select the one below that is the best fit for your research. If you are not sure, read the appropriate sections before making your selection.

☒ Life sciences ☐ Behavioural & social sciences ☐ Ecological, evolutionary & environmental sciences

For a reference copy of the document with all sections, see nature.com/documents/nr-reporting-summary-flat.pdf

Life sciences study design

All studies must disclose on these points even when the disclosure is negative.

Sample size	Because this study includes different analyses, all details are provided in the methods section.
Data exclusions	No exclusions. All data are included in the article and its supplementary information.
Replication	The reproducibility of enzyme kinetics was tested at least 2-3 times with freshly prepared enzyme and stock solutions of the substrate and inhibitor. Once this was determined to provide consistent reaction rates within an error limit of 5%, the final experiment for the data displayed in the manuscript was carried out in duplicate and 4 reads per well for each time point. The mean of the data points was used for fitting. The same stock solutions of enzyme and inhibitor were used for SV-AUC and ITC analyses to determine the dimer dissociation constant (Kdimer) and the binding constant of the inhibitor to the enzyme (Kd), respectively. Kdimer, Kd and molecular mass were determined with multiple protein constructs and concentrations. Each ITC experiment was carried out with a minimum of 20 injections. The apparent dimer dissociation constants were determined by Lamm equation modeling of the absorbance and interference data. X-ray diffraction data and refinement statistics are shown. Gel images are best representative for each of the construct analyzed.
Randomization	n/a
Blinding	n/a

Reporting for specific materials, systems and methods

We require information from authors about some types of materials, experimental systems and methods used in many studies. Here, indicate whether each material, system or method listed is relevant to your study. If you are not sure if a list item applies to your research, read the appropriate section before selecting a response.

Materials & experimental systems

n/a	Involved in the study
☒	☐ Antibodies
☒	☐ Eukaryotic cell lines
☒	☐ Palaeontology and archaeology
☒	☐ Animals and other organisms
☒	☐ Human research participants
☒	☐ Clinical data
☒	☐ Dual use research of concern

Methods

n/a	Involved in the study
☒	☐ ChIP-seq
☒	☐ Flow cytometry
☒	☐ MRI-based neuroimaging
